# ATP depletion plays a pivotal role in self‐incompatibility, revealing a link between cellular energy status, cytosolic acidification and actin remodelling in pollen tubes

**DOI:** 10.1111/nph.18350

**Published:** 2022-07-23

**Authors:** Ludi Wang, Zongcheng Lin, José Carli, Agnieszka Gladala‐Kostarz, Julia M. Davies, Vernonica E. Franklin‐Tong, Maurice Bosch

**Affiliations:** ^1^ Institute of Biological, Environmental and Rural Sciences (IBERS) Aberystwyth University Plas Gogerddan Aberystwyth SY23 3EE UK; ^2^ Key Laboratory of Horticultural Plant Biology Huazhong Agricultural University Wuhan 430070 China; ^3^ Department of Plant Sciences University of Cambridge Cambridge CB2 3EA UK; ^4^ School of Biosciences, College of Life and Environmental Sciences University of Birmingham Edgbaston Birmingham B15 2TT UK

**Keywords:** actin cytoskeleton, Arabidopsis, ATP, cytosolic acidification, *Papaver rhoeas*, pH, pollen tubes, self‐incompatibility

## Abstract

- Self‐incompatibility (SI) involves specific interactions during pollination to reject incompatible (‘self’) pollen, preventing inbreeding in angiosperms. A key event observed in pollen undergoing the *Papaver rhoeas* SI response is the formation of punctate F‐actin foci.
- Pollen tube growth is heavily energy‐dependent, yet ATP levels in pollen tubes have not been directly measured during SI. Here we used transgenic Arabidopsis lines expressing the *Papaver* pollen *S*‐determinant to investigate a possible link between ATP levels, cytosolic pH ([pH]_cyt_) and alterations to the actin cytoskeleton.
- We identify for the first time that SI triggers a rapid and significant ATP depletion in pollen tubes. Artificial depletion of ATP triggered cytosolic acidification and formation of actin aggregates. We also identify *in vivo*, evidence for a threshold [pH]_cyt_ of 5.8 for actin foci formation. Imaging revealed that SI stimulates acidic cytosolic patches adjacent to the plasma membrane.
- In conclusion, this study provides evidence that ATP depletion plays a pivotal role in SI upstream of programmed cell death and reveals a link between the cellular energy status, cytosolic acidification and alterations to the actin cytoskeleton in regulating *Papaver* SI in pollen tubes.

## Abstract 2

See also the Commentary on this article by Cheung, **236**: 1625–1628.

## Introduction

Flowering plants use complex pollen–pistil interactions that play a decisive role in determining reproductive success (Johnson *et al*., 2019). Self‐incompatibility (SI) is a genetically controlled pollen–pistil recognition system regulated by tightly linked polymorphic *S*‐determinant genes expressed in the pollen and pistil. These *S*‐determinants define mating types and prevent potentially deleterious inbreeding. In *Papaver rhoeas*, the *S*‐determinants are PrsS (a secreted signalling ligand, related to cysteine‐rich proteins) and PrpS, a small transmembrane protein (Foote *et al*., 1994; Wheeler *et al*., 2009). We have demonstrated the functional transfer of the *Papaver* SI system to Arabidopsis; co‐expression of cognate *PrpS* and *PrsS* in self‐compatible *Arabidopsis thaliana* makes these plants self‐incompatible (de Graaf *et al*., 2012; Lin *et al*., 2015).

Analysis of events downstream of the interaction of cognate *S*‐determinants in *Papaver* has shown that this involves a Ca^2+^‐dependent signalling network triggered by SI, involving several components and targets that result in programmed cell death (PCD; Thomas & Franklin‐Tong, 2004; Wang *et al*., 2018). One key target is the actin cytoskeleton. In incompatible pollen tubes, after SI induction, dramatic remodelling was observed: the distinctive longitudinal actin filament (F‐actin) bundles disappear, accompanied by a large reduction in actin polymer level (Snowman *et al*., 2002). Actin subsequently reorganizes into large, highly stable actin foci (Geitmann *et al*., 2000; Poulter *et al*., 2010, 2011). The colocalization of the actin binding proteins (ABPs) villin, CAP and ADF with the punctate actin foci (Poulter *et al*., 2010; Zhao *et al*., 2020), suggests their potential role in mediating this remodelling. Increases in reactive oxygen species (ROS) and cytosolic acidification play a pivotal role in mediating the formation of the actin foci (Wilkins *et al*., 2011, 2015). These studies largely relied on using fixed *Papaver* pollen tubes combined with phalloidin staining of F‐actin, so detailed information on the F‐actin dynamics stimulated by SI is lacking. We recently described transgenic Arabidopsis lines expressing PrpS in the pollen together with Lifeact‐mRuby2 (Wang *et al*., 2020), which enables live‐cell imaging of actin.

The actin cytoskeleton is a complex dynamic network that undergoes rapid assembly and disassembly in response to various cues. Actin filament formation requires constant energy consumption in the form of adenosine 5′‐triphosphate (ATP). Although ATP hydrolysis is not essential for polymerization to occur, it is required for treadmilling and maintenance of actin filaments with actin acting as an ATPase. It is thought that global treadmilling exists for all actin networks within the cell (Carlier & Shekhar, 2017). Numerous ABPs are known to regulate actin turnover and changes in configuration (Hussey *et al*., 2006; Staiger & Blanchoin, 2006; Carlier & Shekhar, 2017). Although actin has long been established as being crucial in regulating pollen tube growth, the cellular mechanisms underlying regulation of actin during pollen tube growth are still being uncovered. Pollen tubes have distinctive tip‐localized cytosolic free Ca^2+^ ([Ca^2+^]_cyt_) and H^+^ gradients; these influence actin dynamics and organization (Xu & Huang, 2020).

ATP acts as a universal cellular energy cofactor fuelling all life processes, including protein synthesis, deacylation reactions, metabolism and transport. ATP is produced either by using oxidative phosphorylation in the mitochondria or by the glycolytic pathway. The current consensus is that in plant cells, cytosolic ATP is provided mainly by the mitochondria (De Col *et al*., 2017). Pollen tube growth is a higher ATP‐consuming process than vegetative growth; see (Rounds *et al*., 2011) for a review. Although ATP produced from the mitochondrial respiratory chain is a major source of energy during pollen tube growth, aerobic fermentation also plays an important role (Rounds *et al*., 2011; Obermeyer *et al*., 2013). Artificial inhibition of the mitochondrial electron transport chain in pollen tubes results in a rapid rearrangement of metabolic pathways with ethanol fermentation compensating the reduced ATP production by oxidative phosphorylation (Obermeyer *et al*., 2013). In animal cells, energy starvation at the cellular level elicits diverse responses, which often are ancient, conserved and, importantly, decide whether a cell survives, becomes quiescent, or dies (Buelto & Duncan, 2014). In plants, there are fewer reports of ATP depletion, but it has been observed during various forms of PCD (Tiwari *et al*., 2002; Krause & Durner, 2004; Hatsugai *et al*., 2012), suggesting that lack of ATP may be an early signal to trigger PCD. ATP levels in pollen tubes have not been directly measured during the SI response, however, we recently identified irreversible oxidation of ATP synthesis and metabolic enzymes after SI induction (Haque *et al*., 2020), which could potentially affect the cellular energy status.

Cytosolic pH (pH_cyt_) plays a crucial role in pollen tube growth and must be closely regulated. Under normal cellular conditions, the pH_cyt_ is dynamic; pollen tubes normally have a longitudinal tip to shank pH_cyt_ gradient. The pH_cyt_ at the tip oscillates but is always more acidic than the shank region; it can be up to 1 pH unit lower at the tip (Feijó *et al*., 1999; Michard *et al*., 2009; Hoffmann *et al*., 2020). The acidic tip is sustained by H^+^ efflux along the pollen tube shank (due to the activity of plasma membrane (PM) H^+^‐ATPases in that region) and H^+^ re‐entry at the apex (Hoffmann *et al*., 2020). We previously demonstrated that SI triggers a very large decrease in pH_cyt_ to pH 5.5 in pollen tubes of both *Papaver* (Bosch & Franklin‐Tong, 2007; Wilkins *et al*., 2015) and Arabidopsis co‐expressing PrpS and a pH‐sensitive ratiometric GFP (pHGFP; Wang *et al*., 2020). As many cellular processes are regulated by pH, this dramatic acidification will have consequences for cellular function, including enzyme activity. Indeed, cytosolic acidification triggers the formation of punctate actin foci in *Papaver* pollen tubes (Wilkins *et al*., 2015); this remodelling probably involves several ABPs, which presumably have their activity altered by SI signals. A possibility that is often overlooked is that pH itself can affect actin dynamics. Biophysical studies of actin dynamics *in vitro* show that pH can indeed have a significant effect on actin polymer dynamics (Wang *et al*., 1989; Arii & Hatori, 2008; Crevenna *et al*., 2013; Wioland *et al*., 2019).

Here we demonstrate that SI triggers rapid, significant ATP depletion in incompatible pollen tubes and provide evidence that this results in a sharp drop in pH_cyt_ and later, formation of actin aggregates. This implicates for the first time that ATP depletion is likely to be a central player, pivotal to SI responses. Moreover, visualization of SI‐induced localized dynamic acidic patches adjacent to the PM implicates cytosolic H^+^ accumulation as a very early SI event.

## Materials and Methods

### Plant material, growth conditions and treatments

Transgenic *Arabidopsis thaliana* lines, coexpressing *PrpS* and markers (Supporting Information Table S1) were used. For actin imaging, a ‘rapid’ and a ‘slow’ line expressing Lifeact‐mRuby2 were used (Table S1; de Graaf *et al*., 2012; Wang *et al*., 2020). For imaging pH_cyt_, plants co‐expressing a pHGFP reporter (Table S1; Wang *et al*., 2020) were used. Plants were grown to flowering at 22°C (16 h : 8 h, light : dark) and pollen grains harvested from mature (stage 13) flowers. Arabidopsis pollen tubes were grown *in vitro* in liquid growth medium (GM), pH 7.0, as described previously (Wang *et al*., 2020), for > 60 min before live‐cell imaging. *Papaver rhoeas* pollen from field‐grown plants was collected and stored over silica gel at −20°C and grown *in vitro* in PrGM, pH 6.8, as described previously (Snowman *et al*., 2002).

For SI induction, recombinant PrsS_1_ proteins were produced and dialysed as described by Wilkins *et al*. (2015) or Lin *et al*. (2020) for roots. Self‐incompatibility was induced by adding PrsS_1_ to pollen from the Arabidopsis lines or PrsS_1_ and PrsS_3_ to *Papaver* pollen tubes growing in GM (final concentration 20 μg ml^−1^). For experiments using Good's buffer, GM was replaced with GM in 50 mM PIPES buffer, pH 7.0 immediately before SI induction. Cytosolic pH was manipulated using propionic acid buffer as described previously (Wilkins *et al*., 2015). ATP depletion was induced by adding 10 μM antimycin A and 15 mM 2‐deoxy‐d‐glucose (Sigma‐Aldrich).

### Imaging and image analyses of actin

For imaging and measurements of F‐actin in pollen tubes and actin filaments *in vitro*, time‐lapse images of pollen tubes growing *in vitro* were captured using a Leica DMi8 microscope equipped with a Leica TCS SPE camera. Fiji was used for image processing and quantification (Schindelin *et al*., 2012). For viability staining, pollen tubes were treated with 2 μM propidium iodide (PI) and fluorescence observed (Leica DMi8). F‐actin labelled with Lifeact‐mRuby2 was observed using a Leica SP8 confocal microscope (×100 CS2 objective, NA 1.40, ex. 561 nm, em. 575–750 nm). Signal‐to‐noise ratio was improved using ‘Subtract Background’ (radius of 50 pixels). *Z*‐stack time‐lapse images of F‐actin were thresholded to generate binary images that were skeletonized (Process‐Binary‐Skeletonize) and full projections were used for quantification of ‘occupancy’, ‘average fragment lengths’ and ‘density of end points’ (Zhao *et al*., 2020). The 0–25 μm from the pollen tube tip was selected as the region of interest (ROI). The ROI average fragment length and end points were obtained using the ‘Analyse Skeleton’ tool (Arganda‐Carreras *et al*., 2010); end point density was calculated by dividing end point number by ROI area (μm^2^). Shortening rates of F‐actin bundles were calculated by dividing the difference in length by the time intervals. Only actin filament cables that continuously shortened for > 10 s were analyzed.

### Measurement of cytosolic pH

Imaging of *A. thaliana* pollen tubes expressing pHGFP (Table S1) growing *in vitro* was carried out using a Leica SP8 confocal microscope (×100 CS2 objective, NA 1.40; Wang *et al*., 2020). Images were processed using Lightning deconvolution (Reymann, 2018). Fluorescent intensity ratios R_405/488_ were processed and quantified using Leica Application Suite X (LAS X) software. Calibration of all of the pH values was carried out in untreated pollen tubes using 50 mM propionic acid at different pH. A calibration curve of pHGFP was made after each imaging session by measuring the R_405/488_ at defined pH values (Fig. S1a).

A nigericin pH_cyt_ clamp (Hoffmann *et al*., 2020) was used before and after SI induction to validate the propionic acid *in vivo* [pH]_cyt_ calibration. Elongating pollen tubes expressing pHGFP treated with 30 μM nigericin and 140 mM KCl in 10 mM sodium phosphate buffer at different pH, either before or after SI induction, gave an indistinguishable calibration to the propionic acid method over the pH 5.5–7 range (Fig. S1a). These calibrations verify the [pH]_cyt_ measurements made using propionic acid. Monitoring pH_cyt_ change in *A. thaliana* pollen tubes after SI‐induction using the three different calibration methods all showed acidification to *c*. pH 5.5 (Fig. S1b).

### ATP assays

Arabidopsis pollen from 25–30 flowers or *P. rhoeas* pollen (S_1_S_3_, 2 mg) was grown for 2 h before treatment. Luminescent ATP Detection Assay Kit (ab113849; Abcam, Cambridge, UK) was used to measure ATP levels in pollen tube extracts. Pollen tubes were washed with GM, 50 μl kit detergent added and extraction carried out using the kit protocol by grinding followed by centrifugation (6000 ***g*** for 5 min, 4°C). Substrate was added to samples and luminescence measured after 15 min (Hidex Sense microplate reader; Hidex, Turku, Finland).

### DEVDase activity assays

Arabidopsis pollen from 100 to 130 flowers was germinated and SI was induced. Pollen was collected by centrifugation (6000 ***g***, 5 min). Protein extraction and DEVDase activity assays were carried out as described by Wilkins *et al*. (2011). Fluorophore release by cleavage was measured (ex. 355 nm; em. 460 nm) using a FLUOstar Omega reader (BMG Labtech, Ortenberg, Germany) for 5.5 h at 27°C.

### Measurement of pH_apo_ in Arabidopsis roots

Arabidopsis seedling roots expressing *PrpS* ectopically (Table S1; Lin *et al*., 2020) were used for apoplastic pH (pH_apo_) measurements with HPTS (8‐hydroxypyrene‐1,3,6‐trisulfonic acid trisodium salt; Sigma‐Aldrich), according to Barbez *et al*. (2017). Seedlings were grown for 4 d as described by Lin *et al*. (2020), placed in a 35 mm glass‐bottom microwell dish with a no. 1.5 coverslip (MatTek Corp., Ashland, MA, USA) and dark‐incubated in 1/5 LRC2 liquid medium with 5 mM HPTS ± PrsS_1_ (10 μg ml^−1^). Compatible PrsS_3_ and LRC2 alone acted as controls. Imaging used a Leica SP8 confocal microscope (×100 CS2 objective, NA 1.40), using sequential excitation at 458 and 405 nm, and emission detected between 483 and 563 nm. Fluorescence intensity ratios (R_458/405_) were processed and quantified using Las X. Calibration of pH_apo_ used standard curves generated by *in vitro* calibration of HPTS, according to (Barbez *et al*., 2017).

### Imaging of actin filaments *in vitro*

Labelled rabbit muscle F‐actin filaments were polymerized with equimolar rhodamine phalloidin (4 μM) at 24°C overnight, as described by (Huang *et al*., 2004) with modifications. Polymerized F‐actin was diluted to 10 nM in fluorescence buffer (Huang *et al*., 2004), with 10 mM imidazole at different pHs (7.0, 6.5, 6.0 and 5.5). A 5‐μl sample was applied to a glass‐bottom dish coated with 0.01% (w/v) poly‐l‐lysine and imaging performed after 10 min.

## Results

### Arabidopsis SI‐lines reconstitute the *Papaver* SI‐induced actin alterations

To date, detailed information on F‐actin dynamics stimulated by SI in *Papaver* has been missing, as studies relied on fixed material. The availability of Arabidopsis lines expressing PrpS with LifeAct‐mRuby2 has allowed the first live‐cell imaging analysis of these events (Wang *et al*., 2020). Here we primarily used a ‘rapid’ line (Table S1). Untreated, growing pollen tubes exhibited actin configurations of predominantly longitudinally orientated long actin bundles in the shank, with more dynamic, less bundled shorter actin filaments in the tip region (Fig. S2a). Rapid changes to the actin configuration were observed after SI induction (Fig. S2a; Video S1). Within a few seconds, actin was observed at the pollen tube tip (Fig. S2b). The orientation of actin bundles near the cortex became more disorganized (Fig. S2c). Fragmentation of thick actin filament bundles also was observed (Fig. S2d) concomitant with a reduction in actin bundles (Fig. S2e), followed by the formation of punctate actin foci (Fig. S2f). Compatible pollen tubes did not show actin alterations (Fig. S2g). This essentially describes the same progression and pattern of events to those observed in *Papaver* pollen tubes, providing confidence that we can use these transgenic Arabidopsis pollen tubes to analyze the SI response meaningfully.

### Early SI involves shortening and severing of actin filament bundles

**Fig. 1:**
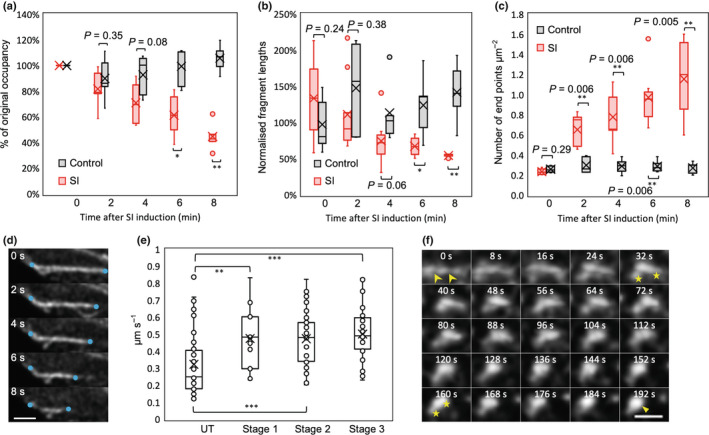
Self‐incompatibility (SI) induces shortening and severing of actin filament bundles and aggregation into foci. (a–c) Quantification of changes to filamentous (F)‐actin in pollen tubes during the early SI response in an *Arabidopsis thaliana* ‘rapid’ line co‐expressing PrpS_1_ and Lifeact‐mRuby2. The mean values are indicated by crosses. Controls comprised pollen tubes treated with growth medium. (a) Quantification of the ‘occupancy’ of F‐actin in pollen tubes; occupancy at time ‘0′ was set at 100%. *, *P* = 0.02; **, *P* = 0.009 (*n* = 5). (b) Quantification of the average fragment lengths of actin filaments; the control at time ‘0′ was normalized to 100%. *, *P* = 0.02; **, *P* = 0.009 (*n* = 5). (c) Quantification of the density of end points of actin filaments. **, *P* < 0.01 (*n* = 5). (d) Time‐lapse images showing a representative example of shortening of F‐actin cables in a ‘slow’ line co‐expressing PrpS_1_‐GFP and Lifeact‐mRuby2. Series of time‐lapse images were captured at 2‐s intervals within a 30‐s time‐window. Blue dots indicate where evidence of shortening is observed. Bar, 2 μm. (e) Quantification of shortening rates during the first three stages (see Supporting Information Fig. S4) of F‐actin remodelling after SI induction in a ‘slow’ line. Shortening rates were significantly higher after SI induction when compared with untreated (UT) samples; **, *P* = 0.007; ***, *P* < 0.001. Shortening rates did not significantly differ between stages 1, 2 and 3 (*n* = 44, 17, 38, 34 for UT and SI stages 1, 2, 3, respectively). (f) Time‐lapse images showing formation of F‐actin foci in a ‘rapid’ line during the period 5 min 32 s – 8 min 44 s after SI induction (time stamps 0–192 s). Fragmentation of an actin filament bundle (arrow heads at 0 s) was followed by aggregation of fragments (indicated by a pair of stars at 32 and 160 s) into a larger punctate structure (triangle, 192 s). Bar, 1 μm. *P*‐values were obtained with Kruskal–Wallis ANOVA on ranks. For (a–c) the comparisons are with the controls at each time point; for (e) the comparison was with the untreated samples. The central box of the boxplots (a–c, e) shows the central 50% of values (from the first quartile to the third quartile) whereas the whiskers indicate that the values located within 1.5 times the interquartile range. The median is represented as a horizontal line in the central box and the mean is indicated by a cross. Outliers are shown as closed circles (a–c); open circles in (e) represent individual data points.

We examined the ‘occupancy’ of F‐actin, which indicates the level of depolymerization of actin filaments (Higaki *et al*., 2010; Zhao *et al*., 2020), during early SI in a ‘rapid’ line using skeletonized images (Fig. S3). After SI induction, the ‘occupancy’ decreased to 45% of the original level by 8 min (*P* < 0.01; Fig. 1a); there was no change in untreated pollen tubes (*P* = 0.64; Fig. 1a). This confirms SI‐induced actin depolymerization measured in *Papaver* pollen tubes (Snowman *et al*., 2002), lending confidence that these observations reflect authentic *Papaver* SI‐stimulated events. To analyze severing, we quantified actin fragment lengths and the number of end points of actin cables (Arganda‐Carreras *et al*., 2010). Although untreated pollen tubes had variable fragment lengths, reflecting actin turnover, mean lengths were similar (*c*. 0.6 ± 0.06 μm; Fig. 1b). After SI induction, fragment lengths decreased by *c*. 50% within 8 min (*P* = 0.009; Fig. 1b). Analysis of the density of end points revealed that after SI‐induction there was a large (> 500%) increase over the sampling period (Fig. 1c), and little change in untreated pollen tubes (*P* = 0.84, ns; Fig. 1c). These data provide evidence for extensive F‐actin severing stimulated during early SI.

We next analyzed shortening rate, which gives an indication of actin depolymerization (Henty‐Ridilla *et al*., 2013). For this, we used pollen tubes from a ‘slow’ line with lower PrpS_1_ expression levels (Wang *et al*., 2020); see Table S1, with a slower SI response, allowing easier visualization of SI‐induced shortening of actin filaments (Fig. 1d). To enable comparison between these lines, we identified key ‘stages’: at stage 1, actin configurations resembled untreated pollen tubes; at stages 2–3 large‐scale actin breakdown and remodelling was evident; and stage 4 exhibited large actin foci (Fig. S4). During the first three stages, the shortening rate changed from a mean rate of 0.32 μm s^−1^ in untreated pollen tubes to a rate of 0.5 μm s^−1^ in SI‐induced pollen tubes (Fig. 1e). Despite the apparent similarity of actin configurations in untreated and stage 1 pollen tubes (Fig. S4), quantification revealed that shortening rate was already significantly higher in stage 1 pollen tubes compared to untreated pollen tubes (*P* = 0.007). This remained high during stages 2 and 3 (*P* < 0.001 for both), suggesting rapid actin depolymerization; once this starts, the rate remains similar as SI progresses.

### SI‐Induced punctate F‐actin foci are formed by aggregation of small actin fragments

The formation of stable punctate F‐actin foci is one of the most striking events observed during the *Papaver* SI response (Geitmann *et al*., 2000; Snowman *et al*., 2002; Poulter *et al*., 2010). Imaging of Lifeact‐mRuby2 in pollen tubes from the ‘rapid’ line revealed that a striking feature was the speed of formation of punctate foci (Figs 1f, S2f; Video S1). Typically, in SI‐induced pollen tubes, aggregation resulted in formation of foci that merged into a larger structure (Fig. 1f). Most actin foci (81%) were formed in this way, from short F‐actin fragments that aggregated (Fig. S5a). We occasionally observed severing of thick actin cables and these fragments aggregated, forming small foci that subsequently coalesced into larger foci (Fig. S5b,c). Thus, the SI‐induced F‐actin foci form predominantly through aggregation of small fragments of F‐actin bundles at *c*. 10–12 min post‐SI in the ‘rapid’ line.

### SI induces ATP depletion in pollen tubes

**Fig. 2:**
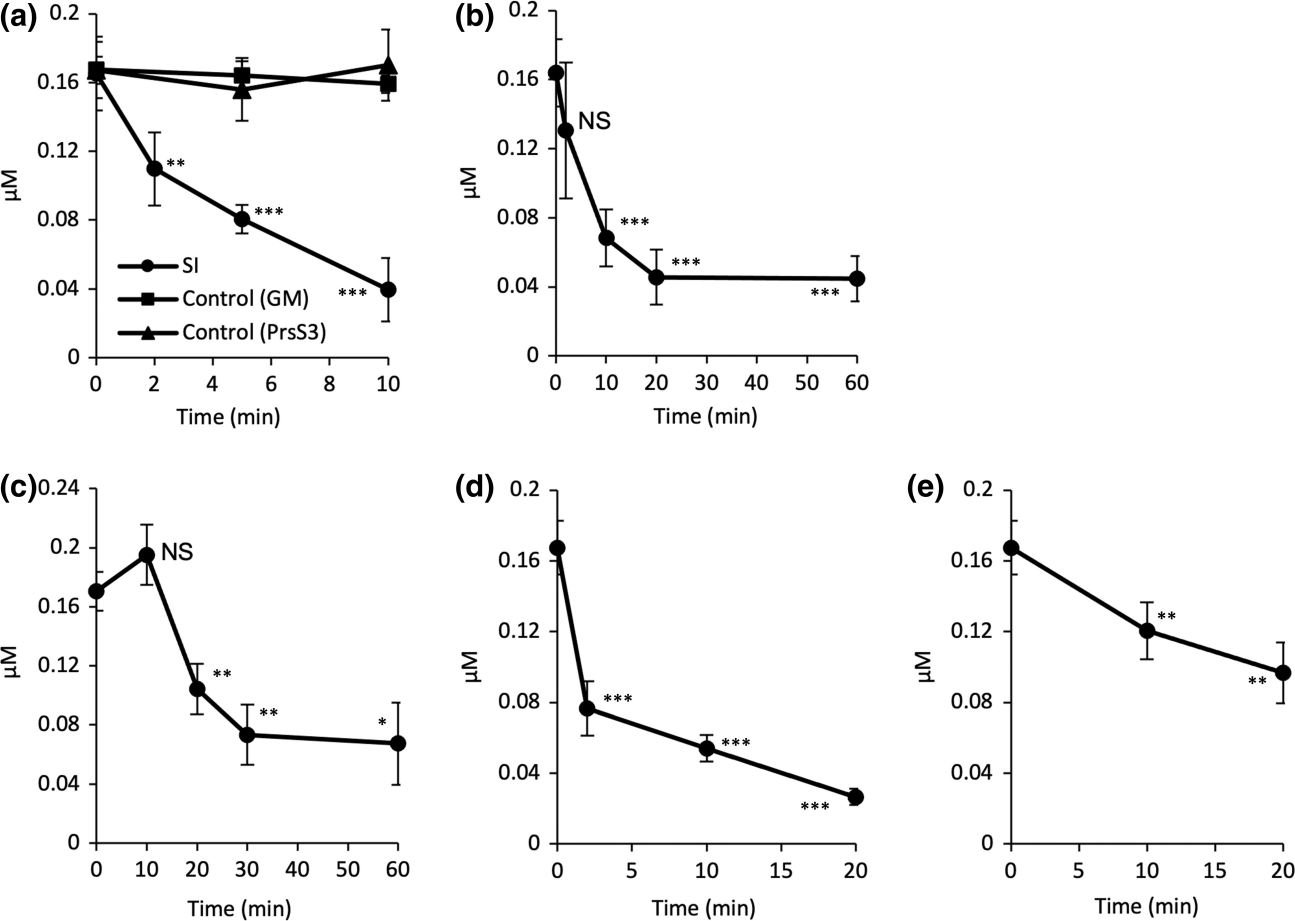
Self‐incompatibility (SI) induces rapid ATP depletion in pollen tubes. (a) Quantification of ATP levels in pollen tubes of an *Arabidopsis thaliana* ‘rapid’ line co‐expressing PrpS_1_ and Lifeact‐mRuby2 after SI induction using recombinant PrsS_1_; controls comprised treatment with growth medium (GM) and compatible PrsS (PrsS_3_; *n* = 3). Two‐way ANOVA shows a significant decrease in ATP within 10 min of SI induction compared with growth medium (GM)‐treatment (*P* < 0.001). One‐way ANOVA between 0 and 2 min of SI induction: **, *P* = 0.002; between 0 and 5 min, and 0 and 10 min, respectively: ***, *P* < 0.001. ATP levels after the treatment with GM or PrsS_3_ did not change significantly (*P* = 0.68 and 0.57, respectively, one‐way ANOVA). (b) Quantification of ATP levels in an *A. thaliana* ‘slow’ line expressing PrpS_1_‐GFP and Lifeact‐mRuby2 after SI induction (*n* ≥ 3). One‐way ANOVA between 0 and 2 min: *P* = 0.17 (ns, not significant); between 0 and 10, 20, 60 min, respectively: ***, *P* < 0.001; between 20 and 60 min: *P* = 0.95. (c) Quantification of ATP levels in *Papaver rhoeas* (haplotype *S*_*1*_ *S*_*3*_) pollen tubes after SI induction (*n* = 3). One‐way ANOVA between 0 and 10 min: *P* = 0.17 (ns); between 0 and 20 min: **, *P* = 0.007; between 0 and 30 min: **, *P* = 0.004; between 0 and 60 min: *, *P* = 0.01; between 30 and 60 min: *P* = 0.78. (d) Treatment of *A. thaliana* ‘rapid’ line pollen tubes with ATP depletion drugs (15 mM of 2‐deoxyglucose (2‐DG) and 10 μM of antimycin A) resulted in rapid ATP depletion (*n* = 3). One way ANOVA between 0 and 2, 10, 20 min, respectively: ***, *P* < 0.001. (e) Treatment of *A. thaliana* ‘rapid’ line pollen tubes with 10 μM of the calcium ionophore A23187 resulted in ATP depletion (*n* ≥ 3), but not to such a low level as SI. One way ANOVA between 0 and 10 min: **, *P* = 0.006; between 0 and 20 min: **, *P* = 0.005. All time points given for a–e depict the point of extraction. All error bars indicate ± SD.

As actin turnover/dynamics are heavily energy‐dependent and require ATP, we investigated whether the intracellular ATP levels ([ATP]_i_) were altered after SI induction in the Arabidopsis lines. [ATP]_i_ was significantly decreased within 10 min of SI induction in the ‘rapid’ line (*P* < 0.001), compared with GM controls; no significant changes in [ATP]_i_ were observed after compatible treatment (*P* = 0.89). Within 2 min of SI induction, [ATP]_i_ decreased to 66% of the original level (Fig. 2a). By 10 min, [ATP]_i_ had fallen to 0.04 μM (± 0.02), 24% of its original level. The [ATP]_i_ in the controls did not change significantly during this period (*P* = 0.68 and 0.57, respectively), remaining *c*. 0.16 μM (Fig. 2a). In the ‘slow’ line [ATP]_i_ was reduced by 73% after 20 min and remained low for at least 60 min (Fig. 2b). This demonstrates that SI triggers rapid and significant ATP depletion in incompatible pollen tubes. To verify that this ATP depletion is an authentic SI‐triggered event, we induced SI in *P. rhoeas* pollen tubes growing *in vitro* and measured [ATP]_i_ in the same way. In *P. rhoeas* pollen tubes, the SI response was slower, but by 20 min, [ATP]_i_ was significantly reduced to 0.10 μM (± 0.01, *P* < 0.01; Fig. 2c). By 30 min, [ATP]_i_ was further reduced by 30% to 0.07 μM (± 0.03) and remained low. Like the response in Arabidopsis pollen, ATP depletion was partial; even after 60 min, ATP levels remained at *c*. 40% of those at *t* = 0.

We next artificially depleted ATP. Treatment of pollen tubes from the Arabidopsis lines growing *in vitro* with 10 μM antimycin A, which inhibits mitochondrial electron transport, and 15 mM 2‐deoxyglucose (2‐DG), which blocks glycolysis, effectively induced ATP depletion to similar levels as SI. A 54% reduction in ATP levels was observed within 2 min, from 0.17 μM (± 0.02) to 0.08 μM (± 0.02) with a further decrease to 16% of the initial level by 20 min (Fig. 2d). The extent of the decrease and time course matched the levels of ATP depletion triggered by SI closely. Growth arrest is a key feature of SI. The ATP depletion drugs led to almost instantaneous growth arrest, within 1 min, a similar time frame as observed for SI in the ‘rapid’ line (Video S2; Fig. S6). Thus, a drop in [ATP]_i_ similar to that observed early in SI is sufficient to inhibit growth. As other SI‐induced events can inhibit tip growth (e.g. increases in [Ca^2+^]_cyt_), we examined the effect of the Ca^2+^ ionophore A23187 on [ATP]_i_. This also triggered ATP depletion, but not to such a low level as SI (Fig. 2e), demonstrating that increases in [Ca^2+^]_cyt_, which is the first event observed after SI induction (Franklin‐Tong *et al*., 1993), triggers ATP depletion; however, this ionophore also can permit H^+^ influx (Jyothi *et al*., 1994).

### ATP Depletion induces remodelling of the actin cytoskeleton in pollen tubes

**Fig. 3:**
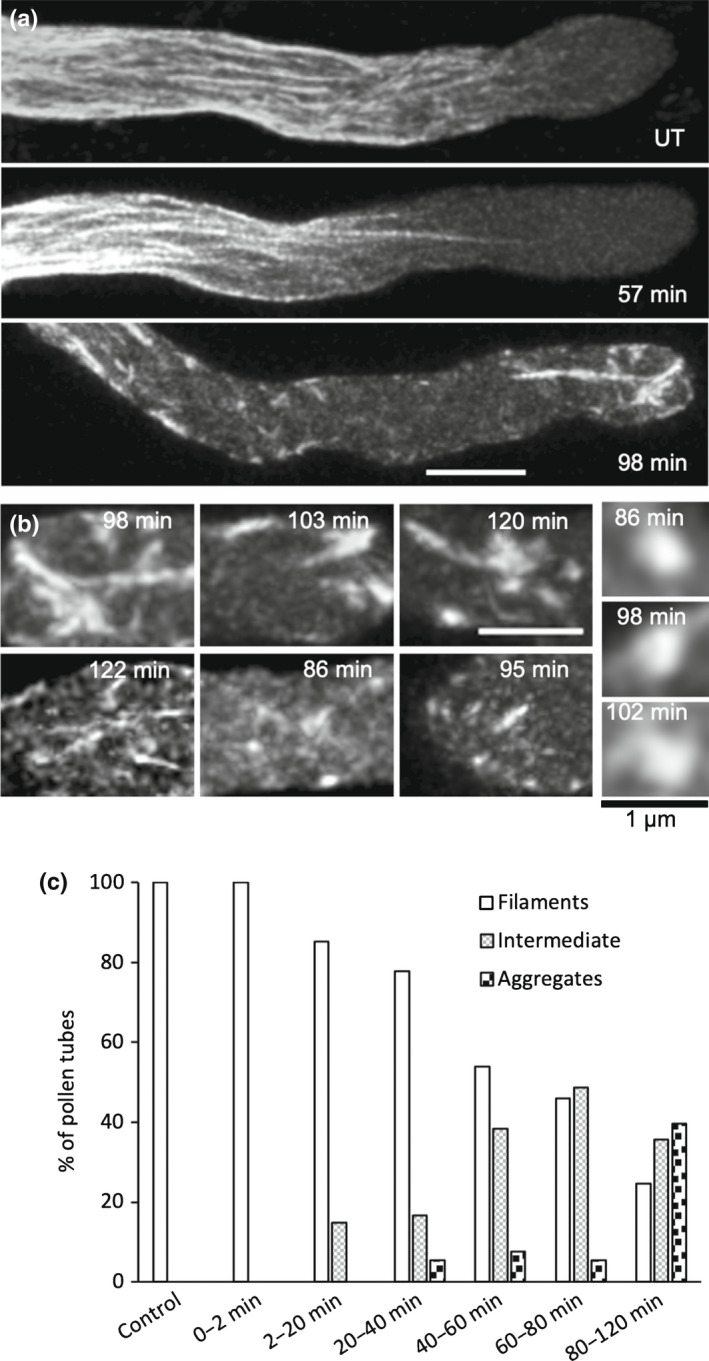
ATP depletion induced in pollen tubes by 2‐deoxyglucose (2‐DG) and antimycin A results in very late formation of actin aggregates. ATP depletion was induced by treating *Arabidopsis thaliana* ‘rapid’ line pollen tubes expressing PrpS_1_ and Lifeact‐mRuby2 with 15 mM 2‐DG and 10 μM antimycin A. Actin configuration was visualized using Lifeact‐mRuby2. Time‐stamps indicate the time after treatment. (a) Representative images showing actin configurations after the ATP depletion treatment; UT, untreated. Bar, 5 μm. (b) Examples of typical actin aggregates formed after ATP depletion. Bars, 3 μm (zoomed in images on the right, 1 μm). (c) Percentage of pollen tubes showing three actin configuration categories (filaments, intermediate, and aggregates) after ATP depletion treatment. *n* = 83 (Control), 11 (0–2 min), 27 (2–20 min), 36 (20–40 min), 39 (40–60 min), 37 (60–80 min) and 73 (80–120 min).

In order to determine whether ATP depletion plays a role in mediating the SI‐induced actin alterations, we treated pollen tubes expressing LifeAct‐mRuby2 (Table S1) growing *in vitro* with antimycin and 2‐DG and imaged actin. We observed very late actin aggregation, resembling aspects of the SI‐induced actin foci, but long after the ATP concentration dropped (Fig. 3a,b). Quantification revealed that actin aggregates were observed at low frequency in pollen tubes between 20 and 80 min after ATP depletion, with more aggregates observed after 80 min (Fig. 3c). Thus, although ATP depletion affects actin organization, it does not fully mimic SI. This suggests that ATP depletion itself does not affect actin organization, but it may contribute to the later actin aggregation.

### ATP depletion in pollen tubes results in cytosolic acidification

**Fig. 4:**
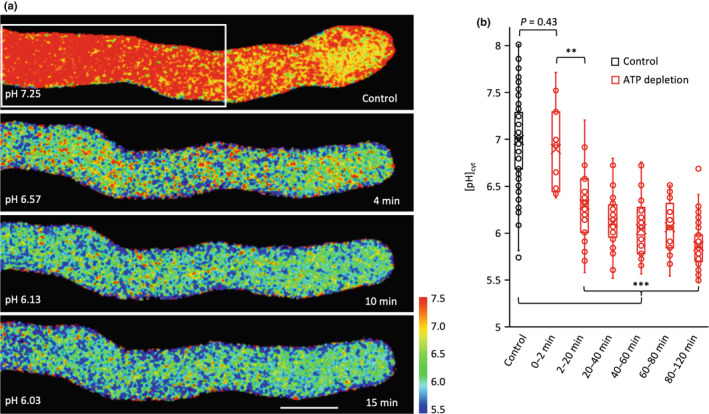
Adenosine 5′‐triphosphate (ATP) depletion induced by 2‐deoxyglucose (2‐DG) and antimycin A results in cytosolic acidification in pollen tubes. ATP depletion was induced by treating *Arabidopsis thaliana* ‘rapid’ line pollen tubes co‐expressing PrpS_1_ and Lifeact‐mRuby2 with 15 mM 2‐DG and 10 μM antimycin A. Cytosolic pH ([pH]_cyt_) was measured by ratio‐imaging of the pH indicator pHGFP, calibrated using propionic acid, in an ROI area 15–35 μm distal from the pollen tube apex (white box in (a)). (a) Representative images showing that the [pH]_cyt_ declines rapidly after addition of the ATP depletion drugs. Bar, 5 μm. The pH value indicated in each image denotes the average pH within the region of interest (ROI). Pseudocolour scale shows calibrated pH values. (b) Quantification of [pH]_cyt_ in pollen tubes at different time periods after ATP depletion reveals a significant reduction in [pH]_cyt_. *P*‐values were obtained with a Kruskal–Wallis ANOVA on ranks (**, *P* = 0.001; ***, *P* < 0.001). *n* = 83 (Control), 11 (0–2 min), 27 (2–20 min), 36 (20–40 min), 39 (40–60 min), 37 (60–80 min) and 73 (80–120 min). The central box of the boxplot shows the central 50% of values (from the first quartile to the third quartile) whereas the whiskers indicate the values located within 1.5 times the interquartile range. The median is represented as a horizontal line in the central box and the mean is indicated by a cross. Open circles depict individual data points.

As ATP depletion can affect intracellular pH (pH_cyt_; Cassel *et al*., 1986), we examined if this was the case in pollen tubes. A typical, representative control pollen tube had a shank pH_cyt_ of 7.25 in a region 15–35 μm behind the tip and treatment of pollen tubes with 2‐DG and antimycin A resulted in a dramatic reduction in pH_cyt_. Distinctive acidic patches adjacent to the PM were observed (Fig. 4a). Quantification of [pH]_cyt_ triggered by ATP depletion revealed a significant drop in mean shank pH_cyt_ in a region 15–35 μm behind the tip from pH 6.99 ± 0.5 in untreated, growing pollen tubes to pH 6.30 ± 0.4 in treated pollen tubes within 2–20 min (*P* < 0.001; Fig. 4b) and the pH_cyt_ dropped further, to pH 5.82 ± 0.2. This demonstrates that ATP depletion results in a large, rapid decrease in pH_cyt_.

### A drop in [pH]_cyt_ stimulates actin filament breakdown and aggregation

**Fig. 5:**
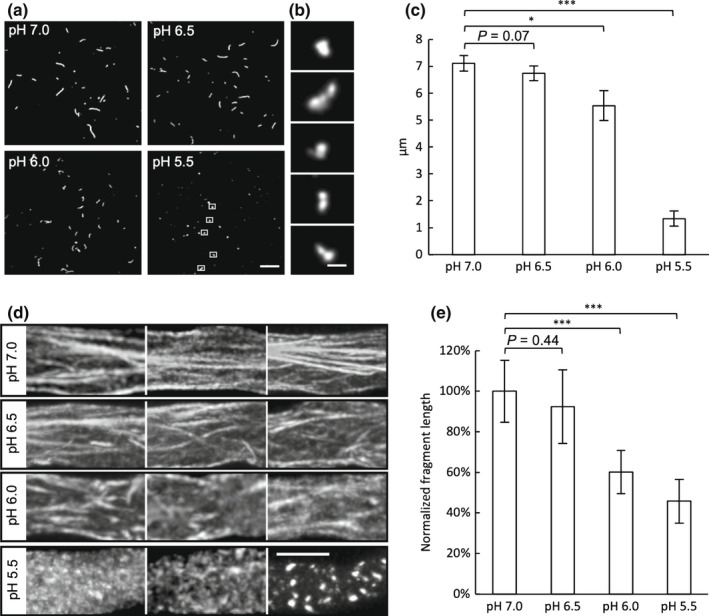
A drop in pH stimulates actin filament breakdown *in vitro* and *in vivo*. (a) Mammalian filamentous (F)‐actin polymerized *in vitro* at pH 8 visualized by fluorescence microscopy after dilution to conditions with lower pH. A reduction in actin filament lengths was observed at reduced pH. Bar, 20 μm. (b) Zoom‐in images indicated by white boxes in (a) show aggregation of actin fragments at pH 5.5. Bar, 2 μm. (c) Quantification of mean lengths of mammalian actin filaments/fragments from slide assays at different pHs. More than 10 individual filaments/ fragments were measured for each assay (*n* = 5 assays). Error bars indicate ± SD. *, *P* = 0.01; ***, *P* < 0.001 (Student's *t*‐test). (d) *Arabidopsis thaliana* pollen tubes expressing PrpS_1_‐GFP and Lifeact‐mRuby2 were treated with propionic acid to manipulate the cytosolic pH ([pH]_cyt_) to 7.0, 6.5, 6.0 and 5.5 and changes in actin visualized. Representative images show shorter actin filaments at lower pHs and aggregates/foci at pH 5.5. Images were taken between 5 and 10 min after adding propionic acid. Bar, 5 μm. (e) Quantification of average fragment lengths of F‐actin in *A. thaliana* pollen tubes expressing Lifeact‐mRuby2 at different pHs. Error bars indicate ± SD (*n* = 6). The average fragment length at pH 7.0 was normalized to 100%. ***, *P* < 0.001 (Student's *t*‐test).

We next examined whether pH itself might contribute to some of the actin remodelling observed during SI. We used an *in vitro* assay using mammalian actin polymerized *in vitro* at pH 8. Imaging the effect of adjusting to more acidic pHs revealed dramatic shortening of actin filament lengths *in vitro* (Fig. 5a). At pH 5.5 (close to the final pH_cyt_ achieved after SI), actin aggregation was apparent (Fig. 5b). Quantitation of F‐actin filament lengths revealed significantly shorter filaments at pH 6.0 (*P* = 0.01) and pH 5.5 compared to controls at pH 7.0 (*P* < 0.001, Fig. 5c). Thus, solely lowering pH *in vitro* (in the absence of ABPs), within the range observed during SI, can stimulate actin filament breakdown and, later, aggregation. This suggests that pH itself could contribute to some of the SI‐induced actin alterations, in concert with ABPs. ADF, CAP and villin are implicated by their co‐localization with the SI‐induced actin foci (Snowman *et al*., 2002; Poulter *et al*., 2010; Zhao *et al*., 2020).

We used an Arabidopsis ‘slow’ line co‐expressing PrpS_1_‐GFP and Lifeact‐mRuby2 to visualize F‐actin remodelling in pollen tubes with [pH]_cyt_ adjusted using propionic acid. Longitudinal actin filament bundles were observed at [pH]_cyt_ 7.0 (Fig. 5d). At reduced pH_cyt_, actin filaments were more disorganized and at [pH]_cyt_ 5.5 fragmented actin and foci were apparent (Fig. 5d). Quantification revealed significantly shorter actin fragments in pollen tubes at [pH]_cyt_ 6.0 and 5.5 (*P* < 0.001; Fig. 5e), compared with pH 7.0. This demonstrates that reduced pH_cyt_ triggers actin bundle fragmentation and confirms that actin foci are formed after actin filament breakdown.

### A threshold cytosolic pH is required for the formation of F‐actin foci

**Fig. 6:**
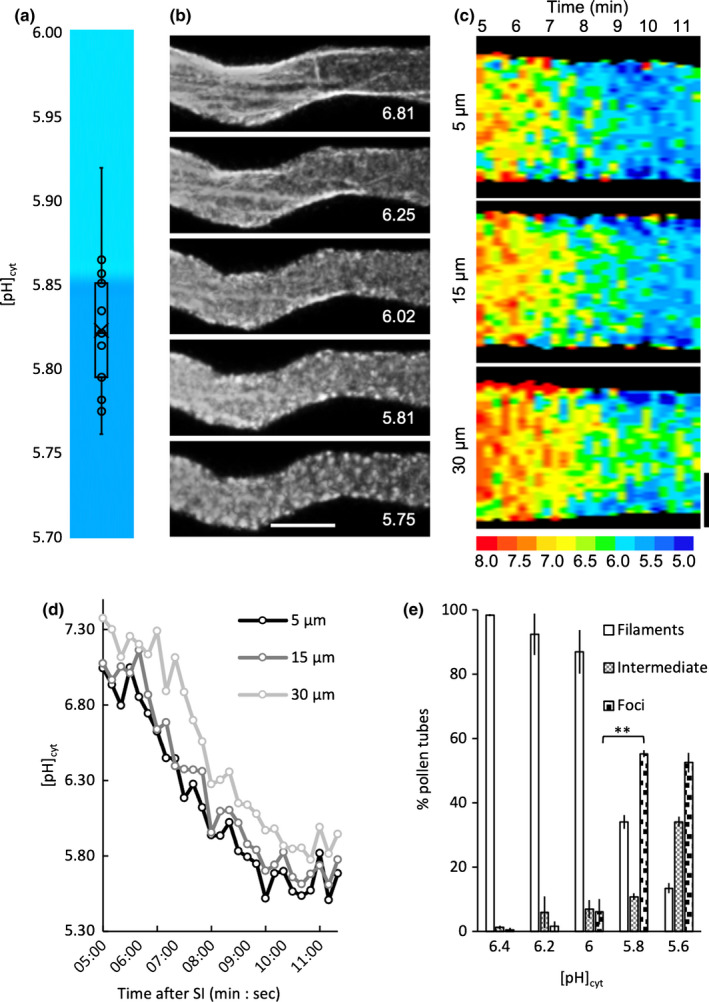
Identification of a cytosolic pH ([pH]_cyt_) threshold for actin foci formation. Pollen tubes from an *Arabidopsis thaliana* ‘rapid’ line co‐expressing PrpS_1_, pHGFP and Lifeact‐mRuby2 were ratio‐imaged to ascertain the [pH]_cyt_ using pHGFP, calibrated by propionic acid, and to monitor actin (using Lifeact‐mRuby2) near‐simultaneously in the same pollen tube. (a) Quantification of the [pH]_cyt_ values (for pseudocolour key see (c)) when actin foci extensively formed in pollen tubes after self‐incompatibility (SI) induction (*n* = 13). pH values correspond to an area of the pollen tube shank region 15–35 μm from the tip. (b) actin configurations in a representative pollen tube after SI induction; the corresponding [pH]_cyt_ value for each image is indicated. Bar, 5 μm. (c) Kymograph analyses of [pH]_cyt_ of a representative pollen tube over a period between 5 and 11.5 min after SI induction (indicated at the top of the kymograph). Three different 5‐μm‐wide regions of the pollen tube were analyzed (centred at 5, 15 and 30 μm from the tip) and reveals spatiotemporal differences in the changes in [pH]_cyt_. The pseudocolour scale shows the calibrated pH values. Bar, 2 μm. (d) Quantification of [pH]_cyt_ within the three 5‐μm‐wide regions of the pollen tube shown in (c) centred at 5, 15 and 30 μm from the tip. This reveals that the region nearer the tip has a lower [pH]_cyt_ than the region behind it and although it goes down, this differential is retained. (e) Percentage of pollen tubes showing three actin configurations (filaments, intermediate and foci) after treatment with propionic acid at different pHs (*n* > 100 per treatment over three independent repeats). Comparison between proportions of pollen tubes with actin foci at pH 6.0 and 5.8: **, *P* = 0.001, Student's *t*‐test. Error bars indicate ± SD.

We used an Arabidopsis ‘rapid’ line co‐expressing pHGFP and Lifeact‐mRuby2 (Table S1) to image the dynamics of pH_cyt_ and F‐actin configuration simultaneously after SI. This revealed that actin foci were initially observed at [pH]_cyt_ 6.02, with large foci detectable when the [pH]_cyt_ reached 5.81 (Fig. 6a,b). Analysis revealed spatiotemporal differences in pH_cyt_, with the region nearer the tip achieving a lower pH_cyt_ earlier (Fig. 6c); pH_cyt_ rapidly decreased from pH *c*. 7.0 to 5.8 between 5 and 9 min after SI induction (Fig. 6d). This suggested that there might be a critical pH_cyt_ ‘tipping point’ at which actin foci formed. To investigate this, we manipulated the pH_cyt_ and determined the pH_cyt_ at which punctate actin foci appeared. Few pollen tubes had actin foci (6% ± 4) at [pH]_cyt_ 6.0, but at [pH]_cyt_ 5.8 a large percentage (55% ± 1) of pollen tubes had actin foci (Fig. 6e). Thus, within this narrow pH range, the number of pollen tubes with foci increased significantly (*P* = 0.001). This suggests a pH_cyt_ threshold of *c*. pH 5.8 for the formation of these F‐actin foci.

### Pollen tubes are not dying during the SI‐ or ATP‐depletion induced acidification time‐period

A possible explanation for the SI‐ or ATP‐depletion induced pH_cyt_ decrease is that the pollen tubes were dying. To determine whether pollen tubes undergoing acidosis were also undergoing PCD at this stage, we examined pollen tube extracts from the Arabidopsis ‘rapid’ line at 20 min after SI induction for caspase‐3‐like/DEVDase activity. This was well after the timepoint when pH_cyt_ plateaued at pH 5.5 and samples had the same DEVDase activity as the untreated controls (*P* = 0.64, ns; Fig. S7a). After artificial ATP depletion using 2‐DG and antimycin, no elevated caspase‐3‐like/DEVDase activity was detected, even after 5 h (Fig. S7b). We also examined SI‐induced pollen tubes for PM integrity. No propidium iodide (PI)‐stained pollen tubes were observed in the period up to 30 min after SI induction (Table S2), demonstrating that the PM was intact within the time frame of the significant pH_cyt_ decrease and providing evidence that SI‐induced pollen tubes were not dying at this early stage of SI. Membrane integrity was lost much later; at 60 min only 10% of pollen tubes were PI‐positive, but by 3 h after SI‐induction 96% were PI‐positive (Table S2). Thus, the SI‐induced ATP depletion and cytosolic acidification observed cannot be a consequence of PCD.

### Evidence for spatially localized pH_cyt_ alterations in SI‐induced pollen tubes

**Fig. 7:**
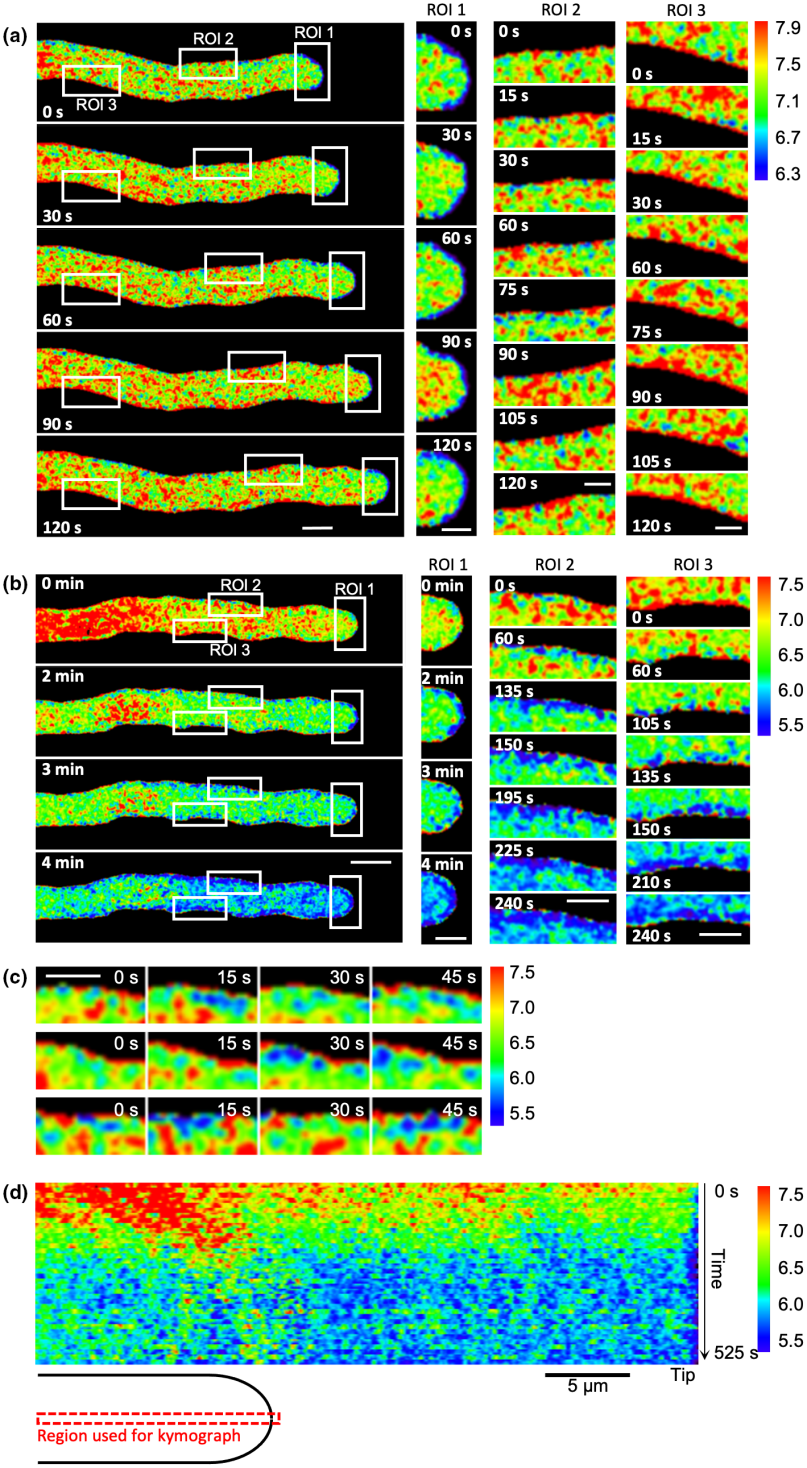
Evidence for localized cytosolic pH ([pH]_cyt_) alterations in self incompatibility (SI)‐induced pollen tubes. Pollen tubes from an *Arabidopsis thaliana* ‘rapid’ line co‐expressing PrpS_1_ and pHGFP (pH calibrated using propionic acid), were ratio‐imaged to ascertain the [pH]_cyt_ in pollen tubes before and after SI induction. The calibrated pseudocoloured [pH]_cyt_ scale and values are shown in the key on the right‐hand side. (a) Set of images from a representative untreated growing pollen tube at various timepoints showing the distribution of [pH]_cyt_ before SI induction (*n* = 15). The pollen tube cytosol is more acidic near the tip, with pronounced localized alkaline patches adjacent to the plasma membrane/apoplast in the shank region. The white boxes indicate three regions of interest (ROI) that are magnified in the right‐hand panels, showing detail. ROI 1 follows the area 0–4 μm from the apex during pollen tube growth. Region of interest 2 follows the area 15–25 μm from the apex during pollen tube growth, whereas ROI 3 indicates an immobilized localization in the shank area. Values of [pH]_cyt_ in different distal areas from the apex are given in Supporting Information Table S3a. Bars: (left‐hand images) 5 μm; (right‐hand (zoomed) images) 2 μm. (b) Set of images from a representative pollen tube after SI induction showing localized regions of acidification adjacent to the plasma membrane (*n* = 23). Note that the pollen tube has stopped growing. The white boxes indicate three regions of interest (ROI) that are magnified in the right‐hand panels. ROI 1 shows the rapid increase in acidic patches at the tip; ROI 2 and 3 show that the localized acidic patches adjacent to the plasma membrane/apoplast are not static, but dynamic. Note that the calibrated pseudocoloured [pH]_cyt_ scale bar is different to that shown in (a); see key on the right. Values of [pH]_cyt_ in different distal areas from the apex are shown in Table S3b. Bars: (left‐hand images) 5 μm; (right‐hand (zoomed) images) 2 μm. (c) Three ROIs showing representative examples of the acidic patches observed adjacent to the plasma membrane (*n* = 20) soon after SI induction in the pollen tube in (b). This revealed that the acidic patches are dynamic as they change their size and position in each image; the time‐stamps indicate time after SI induction at 15 s intervals (0–45 s). Bar, 2 μm. (d) Kymograph analysis of [pH]_cyt_ during the SI response in the representative pollen tube in (b) using a 0.5‐μm‐wide region along the growth axis of the pollen tube (indicated by the cartoon). The pseudocolour scale shows calibrated pH values using propionic acid. Bar, 5 μm.

As we had not previously investigated any spatial aspects of the SI‐induced drop in [pH]_cyt_, we made a more detailed analysis of pH_cyt_ alterations in pollen tubes. Untreated, growing pollen tubes had a longitudinal cytosolic gradient of H^+^ similar to those described in previous studies (Fig. 7a; Table S3a; Video S3). In a representative untreated pollen tube (Fig. 7a) the apical region was more acidic, and acidic patches adjacent to the PM were observed; the mean [pH]_cyt_ in the apical region of pollen tubes varied between 6.65 and 6.90 (*n* = 3; Table S3a). The shank region was more alkaline; the pH_cyt_ of the shank was increasingly alkaline the further the distance from the tip; at 25–35 μm behind the tip the [pH]_cyt_ was between 7.31 and 7.44; (*n* = 3, Table S3a). The more alkaline sub‐apical pH_cyt_ is consistent with H^+^ efflux by plasma membrane H^+^‐ATPase activity (Feijó *et al*., 1999; Certal *et al*., 2008; Hoffmann *et al*., 2020). These observations and measurements of the distribution and gradient of pH measurements of normally growing Arabidopsis pollen tubes using propionic acid‐based calibration of [pH]_cyt_ (see Fig. S1) are similar to those reported by Hoffmann *et al*. (2020).

After SI induction, a drop in pH_cyt_ within 2–3 min and localized distinct, acidic patches were evident adjacent to the PM (Fig. 7b,c; Table S3b; Video S4). The mean [pH]_cyt_ at 0–3 μm from the apex decreased from 6.86 to 5.65 in the first 4 min after SI induction, whereas 25–35 μm from the apex, [pH]_cyt_ decreased from 7.50 to 5.75 (*n* = 3; Table S3b). Critically, sub‐apical regions displayed [pH]_cyt_ values typical of the apex under control conditions within 2–4 min, indicating loss of the normal gradient. The acidic patches adjacent to the plasma membrane (Fig. 7a,c) at both the tip and shank became more pronounced and extensive, forming a peripheral acidic zone in both regions within 135 s (Fig. 7b). These acidic patches were dynamic, changing both in size and location (Fig. 7c; Video S4). Kymograph analysis of the pH_cyt_ along the growth axis provides clear evidence of acidification in the tip region and a progression of acidification over time after SI induction (Fig. 7d). This implies that H^+^ accumulation occurs in both apical and shank regions. These data provide new information about the spatial distribution of pH_cyt_ in pollen tubes and show that major changes in pH_cyt_ occur in incompatible pollen tubes. Our data suggest that H^+^ accumulation is triggered by SI as a very early response in both the tip and shank.

### Evidence for H^+^ influx being triggered by PrpS‐PrsS cognate interaction

**Fig. 8:**
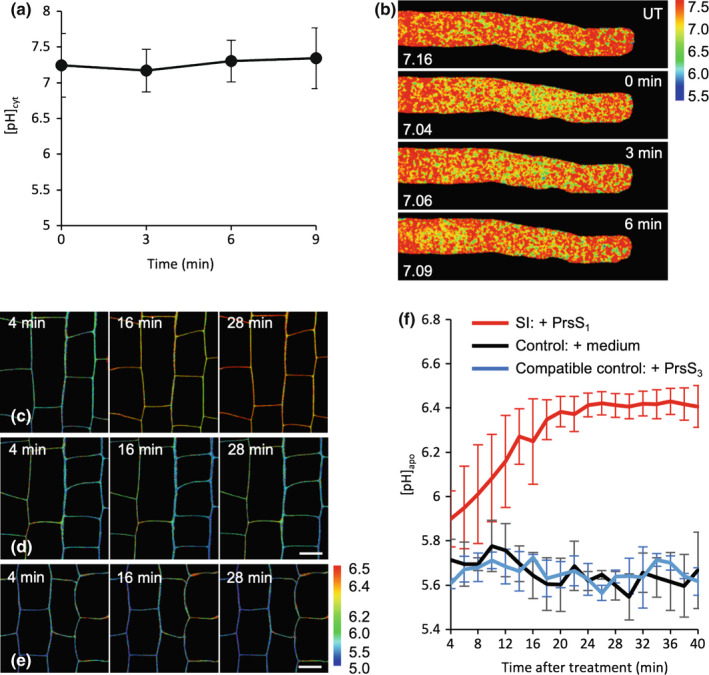
Evidence for H^+^ influx being triggered by PrpS‐PrsS cognate interaction. (a) Quantification of cytosolic pH ([pH]_cyt_) after self‐incompatibility (SI) induction in *Arabidopsis thaliana* pollen tubes co‐expressing PrpS_1_ and pHGFP in growth medium (GM) buffered with 50 mM PIPES, pH 7.0 (*n* = 3). The region of interest (ROI) used for quantification was 15–35 μm from the tip. Error bars indicate ± SD. *P* = 0.94, one‐way ANOVA. (b) Representative images of pollen tubes in GM buffered with PIPES (UT: note that pollen tube growth is inhibited by PIPES and there is no tip‐shank gradient) and after PrsS addition (*t* = 0, 3, 6 min). [pH]_cyt_ values from the ROI are indicated. (c–e) PrsS triggers apoplast alkalinization in Arabidopsis seedling roots expressing cognate PrpS (*pUBQ10::PrpS*_*1*_). Apoplast pH ([pH]_apo_) in basal meristem epidermis‐cortex of 4‐d‐old roots expressing PrpS_1_ was measured by ratiometric imaging of the fluorescent pH indicator 8‐hydroxypyrene‐1,3,6‐trisulfonic acid (HPTS). (c) Representative images showing an increase in [pH]_apo_ after SI induction with PrsS_1_. Pseudocolour scale shows calibrated [pH]_apo_ values. (d) and (e) Representative images of seedling roots after addition of medium (d) and recombinant PrsS_3_ as a compatible control (e). No increase in [pH]_apo_ was detected in these two controls. Bars, 10 μm. (f) Quantification of [pH]_apo_ measured in the imaged area showed a significant increase in [pH]_apo_ after SI induction (*P* < 0.001, two‐way ANOVA comparing [pH]_apo_ after SI induction with controls, error bars indicate ± SD, *n* = 6 for SI induced samples, *n* = 3 for each control group). *P* < 0.001, comparison between [pH]_apo_ at 4 and 24 min after SI induction, Student's *t*‐test. No significant changes in [pH]_apo_ after control treatments, comprising medium (black line, *P* = 0.53) or compatible PrsS_3_ (blue line, *P* = 0.29), one‐way ANOVA.

In order to examine if H^+^ influx might play a role in the cytosolic acidification observed, we buffered the medium and apoplast with a Good's buffer (50 mM PIPES, pH 7) before SI induction in a ‘rapid’ line co‐expressing PrpS_1_ and pHGFP. Good's buffers are membrane‐impermeable, so if SI induces an influx of protons, this should be prevented/delayed. This treatment maintained the pollen tube pH_cyt_ at *c*. pH 7 and SI‐induced cytosolic acidification was prevented (*P* = 0.94, ns; Fig. 8a). Both growth and the apical gradient were abolished by PIPES (Fig. 8b). This provides evidence that extracellular H^+^ contributes to the SI‐induced decrease in pH_cyt_. To investigate this further, we used HPTS to assess apoplastic pH (pH_apo_; Amali *et al*., 2011; Barbez *et al*., 2017). As it was not possible to use pollen tubes because HPTS diffuses into the medium, we used Arabidopsis seedling roots expressing PrpS ectopically (Lin *et al*., 2020) and monitored the pH_apo_ in roots after ‘SI’ induction. Addition of PrsS_1_ to roots expressing PrpS_1_ resulted in an increase in pH_apo_ (Fig. 8c). Control roots did not exhibit altered pH_apo_ (Fig. 8d); likewise, roots expressing PrpS_1_ did not show alkalinization of the apoplast after noncognate PrsS_3_ protein was added (Fig. 8e), demonstrating that this is an *S*‐specific response. Quantification of the pH_apo_ (Fig. 8f) revealed that cognate PrsS addition induced a significant increase in pH_apo_ over a period of 20 min (*P* < 0.001) before reaching a plateau at pH 6.41 (± 0.05) at 24 min. Controls did not exhibit an increase in pH_apo_ (Fig. 8f). As the apoplast is normally maintained at a low pH by H^+^ efflux (Martinière *et al*., 2018; Wegner *et al*., 2021), these data support the idea that H^+^ influx from the apoplast is triggered by SI and provides evidence that this contributes to early SI‐induced cytosolic acidification.

## Discussion

### SI triggers ATP depletion

Here we have established for the first time that SI triggers rapid and dramatic ATP depletion. This suggests that major energetic/metabolic changes are triggered rapidly in incompatible pollen tubes. The speed at which SI‐induction triggers ATP depletion (within a few minutes) is striking. Most reports of ATP depletion in plants describe ATP levels decreasing over several hours (Tiwari *et al*., 2002; Krause & Durner, 2004; Hatsugai *et al*., 2012). Although ATP levels are considerably depleted in SI‐induced pollen tubes, significant ATP levels are retained. This provides evidence that incompatible pollen tubes still produce ATP, albeit, at low levels, and remain alive for several hours before they die.

### How might ATP depletion be achieved and what are the consequences?

ATP is required by living cells for diverse functions. Here, the Ca^2+^ ionophore A23187, which induces key features of SI (Snowman *et al*., 2002; Wilkins *et al*., 2011, 2015) caused ATP depletion. This suggests that [Ca^2+^]_cyt_ elevation, which is an initial step in SI (Franklin‐Tong *et al*., 1993) leads to decreased ATP synthesis through mitochondrial dysfunction and consequent inhibition of oxidative phosphorylation, increased utilization, or efflux from the cells, resulting in altered cellular energy metabolism. One possible scenario is that PrpS itself may act as a Ca^2+^‐permeable or Ca^2+^ and H^+^‐permeable channel protein. Although it has no homologues (Wheeler *et al*., 2009), it appears to be a ‘topological homologue’ of a *Drosophila* protein, Flower, which forms a functional Ca^2+^‐permeable channel (Yao *et al*., 2009; Wheeler *et al*., 2010). Although Flower and PrpS share virtually no primary sequence homology, they share key amino acids in the transmembrane domains that would form a pore (Wheeler *et al*., 2010). The possibility that PrpS may act as a channel needs to be investigated, as do the spatiotemporal relationships between pH_cyt_, [Ca^2+^]_cyt_ and ATP.

Decreases in ATP synthesis usually are caused by mitochondrial defects. Self‐incompatibility in *Papaver* induced rapid release of cytochrome *c* (Thomas & Franklin‐Tong, 2004) and triggered dramatic changes to pollen mitochondrial morphology within 1 h (Geitmann *et al*., 2004). Decreases in ATP synthesis also are associated with damage to mitochondrial proteins by ROS. Self‐incompatibility triggers increases in ROS (Wilkins *et al*., 2011), accompanied by oxidation of many pollen proteins, including a predicted ATP synthase beta subunit (Haque *et al*., 2020), providing a possible mechanism involved in SI‐induced ATP depletion.

Although SI stimulates major ATP depletion, ATP production was not completely inhibited. There are several possible explanations for this. A consequence of ATP depletion often is an increased rate of anaerobic glycolysis, which can maintain cellular energy by generating ATP through metabolism. Pollen tube growth consumes large amounts of ATP (Rounds *et al*., 2011) and it is well‐established that pollen tubes can adapt their metabolic pathways to compensate for reduced mitochondrial ATP production (Rounds *et al*., 2011; Obermeyer *et al*., 2013). Pollen tubes treated with antimycin A had higher glycolytic activity and major changes in pollen metabolism, including dramatic increases in gamma‐aminobutyric acid (GABA; Obermeyer *et al*., 2013). The GABA shunt can alter energy production and it was proposed that the decrease in ATP production by antimycin A reactivates the GABA shunt to generate pyruvate, generating ATP using other metabolic pathways (Kinnersley & Turano, 2000; Fait *et al*., 2008). Thus, there is considerable evidence that pollen tubes alter their metabolism in response to ATP depletion. As we observed dramatic, but incomplete ATP depletion after SI, this suggests that SI switches pollen tubes to glycolytic metabolism. In plants, GABA increases during energetically demanding stresses; it has been suggested that GABA triggered responses help replenish the energetic supply and that this, in parallel with growth arrest, may be pivotal to ensure plant survival under energetically demanding stresses (Michaeli & Fromm, 2015). Examining metabolism after SI and ATP depletion clearly needs to be investigated; future studies should investigate whether SI stimulates GABA production. Moreover, SI triggers inhibition of a soluble inorganic pyrophosphatase (sPPase), p26.1, resulting in increased pyrophosphate (PPi; de Graaf *et al*., 2006; Eaves *et al*., 2017). Increased PPi could provide an alternative energy source. When ATP levels drop in plant cells, PPi can be consumed to ensure that glycolysis continues under conditions of suppressed ATP synthesis, keeping metabolism energy efficient (Igamberdiev & Kleczkowski, 2021). Thus, there are several possible explanations for why, despite ATP depletion, incompatible pollen tubes remain alive.

### How might the observed SI‐induced cytosolic acidification be achieved?

Control of transmembrane pH gradients is a complex interplay of metabolism and H^+^ fluxes (Wegner & Shabala, 2020; Wegner *et al*., 2021). Here, pH gradient disruption arrested growth but did not kill the cells. Pollen tube H^+^ efflux is by plasma membrane electrogenic H^+^‐pumping H^+^‐ATPases (PM H^+^‐ATPases; encoded by *Autoinihibited H* ^*+*^ *‐ATPase* genes, *AHA*s; Fuglsang & Palmgren, 2021). AHAs are responsible for the characteristic spatial H^+^ efflux along the shank of growing pollen tubes that help to generate the longitudinal pH_cyt_ gradient and their activity is required for growth. Application of the Nernst equation indicates that generating the trans‐PM pH gradient of the sub‐apical region of untreated pollen would require such active transporters. At 25–35 μm from the apex, a membrane voltage of +23 mV could cause the observed pH gradient of [pH]_apo_ 7/[pH]_cyt_ 7.40 (Table S3), but PM voltage is far more negative (−127 mV; Mouline *et al*., 2002), indicating the need for energy‐consuming transport afforded by H^+^‐ATPases. In Arabidopsis *aha6/8/9* mutants, H^+^ efflux was lowered; this was accompanied by pH_cyt_ acidification, reduced tip‐to‐shank proton gradients, and defects in actin organization resulting in growth defects (Certal *et al*., 2008; Lang *et al*., 2014; Falhof *et al*., 2016; Hoffmann *et al*., 2020).

Our SI‐induced ATP depletion results imply reduced AHA activity due to restricted ATP supply (known to occur in pollen tubes; Obermeyer & Blatt, 1995), with intracellular acidification (failure to export H^+^) at both apical and sub‐apical regions (Hoffmann *et al*., 2020) as a consequence. Acidosis would further exacerbate the pumps' energy supply as the free energy of ATP declines with declining pH (Davies *et al*., 1993). Our observation of patches of acidic pH_cyt_ adjacent to the PM is consistent with this, the collapse of the normal pH_cyt_ of growing pollen tubes and growth arrest. The pH_cyt_ of the triple *aha* mutant is lowered by 0.5 pH units in the sub‐apical region and PM voltage depolarizes to −70 mV or more positive values (Hoffmann *et al*., 2020). Two other AHAs are found in pollen: AHA1 and AHA7 (Pertl‐Obermeyer *et al*., 2018) so inhibition of further AHAs by ATP depletion could have a more profound effect and help explain the greater pH decrease caused by SI or respiratory inhibition found here (1.5 and 1.2 units, respectively). In the future, it would be useful to determine PM voltage to help discern the contribution of AHA inhibition to cytosolic acidosis.

Plasma membrane H^+^‐ATPase failure could lead to increased net H^+^ influx from the apoplast provided that the H^+^ electrochemical gradient was favourable, and a transport pathway existed. Apoplastic pH typically has a value between 4.8 and 6 (Felle, 2001) and can play an important role in the dynamics of cytosolic acidification (Geilfus, 2017; Wegner *et al*., 2021). Self‐incompatibility‐induced apoplast alkalinization suggests that H^+^ influx may contribute to the decrease in pH_cyt_. The failure in SI‐induced cytosolic acidification when pH_apo_ was buffered to pH 7 (as opposed to unbuffered GM at pH 7) is particularly intriguing, especially given that this collapsed the longitudinal pH_cyt_ gradient under control conditions, inhibiting growth. Buffering pH_cyt_ was found previously to inhibit pollen tube growth (Feijó *et al*., 1999). As H^+^ influx occurs at the apex (Feijó *et al*., 1999; Hoffmann *et al*., 2020), the implication is that extracellular pH (possibly at the extra‐facial PM) must be free to vary below pH 7 to enable normal trans‐PM pH gradients to be generated for growth. That SI‐induced acidosis could not proceed also points to a need for a greater H^+^ concentration at the extra‐facial PM to permit H^+^ influx. Identities of pollen tube H^+^ influx pathways are limited, but the PM H^+^/Cl^−^ symporter TMEM16 transports both H^+^ and Cl^−^ into the sub‐apical cytosol and is a putative pH sensor in pollen (Domingos *et al*., 2019). It would be interesting to determine the impact of loss of TMEM16 on SI‐induced acidosis.

Lowered ATP could have consequences for regulation of several families of transporters that could influence the PM or vacuolar voltage and hence pH_cyt_ (De Angeli *et al*., 2016). Several possible transporters may be relevant for the dynamic changes in pH_cyt_ observed here as SI‐induced acidification progresses. Key amongst these would be the V‐Type H^+^‐ATPases that remove cytosolic H^+^ to acidify organelles. As the vacuolar pH is 5.2, and those of the trans‐Golgi network and multivesicular body are pH 6.3 and 6.2, respectively (Sze *et al*., 2002; Shen *et al*., 2013), these intracellular compartments may contribute transiently to the cytosolic acidification observed after SI as lumenal H^+^ ‘leak’ back down their electrochemical potential gradient (Bronk & Gores, 1991). Acidosis would impair opening of the PM TPK4 K^+^ channel which plays a role in regulating PM voltage, so this might impair the ionic oscillations that are central to pollen tube growth (Becker *et al*., 2014).

### ATP depletion and interplay with events leading to SI‐induced PCD

**Fig. 9:**
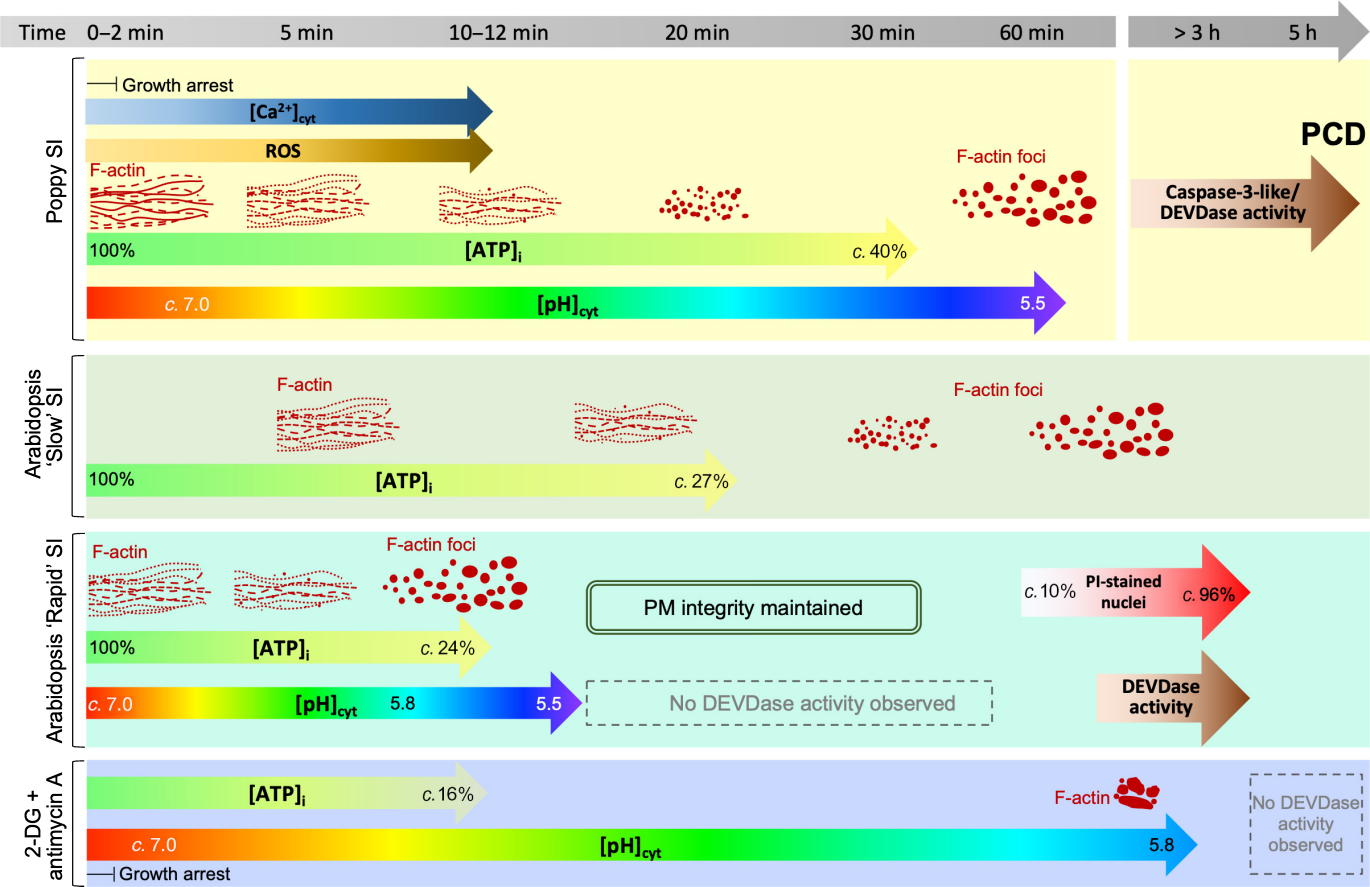
Cartoon of self‐incompatibility (SI)‐ and ATP depletion‐induced events and their timing in *Papaver* and Arabidopsis PrpS‐expressing lines. SI in *P. rhoeas* pollen tubes triggers rapid increases in cytosolic free calcium ([Ca^2+^]_cyt_), reactive oxygen species (ROS) and decreases in cytosolic pH ([pH]_cyt_) which trigger increased caspase‐3‐like/DEVDase activities required for executing programmed cell death (PCD). Filamentous (F)‐actin depolymerization is observed within 10 min, whereas large F‐actin foci are apparent after 60 min. Cytosolic ATP ([ATP]_i_) decreases to 40% after 30 min, whereas [pH]_cyt_ drops to 5.5 after 60 min. In the *Arabidopsis thaliana* ‘slow’ line co‐expressing PrpS_1_‐GFP and Lifeact‐mRuby2, the timing of SI‐induced ATP depletion and actin remodelling shows a similar pattern as in *P. rhoeas* pollen tubes. In the *A. thaliana* ‘rapid’ line co‐expressing PrpS_1_ and Lifeact‐mRuby2, pollen tube [ATP]_i_ is depleted faster; within 10 min after SI induction. Cytosolic acidification occurs rapidly, reaching pH 5.5 after 12 min of SI induction, whereas F‐actin foci form within this timeframe, after a threshold of pH *c*. 5.8 has been reached. Plasma membrane integrity is maintained and no significant increase in caspase‐3/DEVDase activity is detected within the timeframe of dramatic cytosolic acidification and ATP depletion induced by SI, even after [ATP]_i_ and [pH]_cyt_ had plateaued. This demonstrates that PCD is the downstream result and not the cause of SI‐induced ATP depletion and cytosolic acidification. Treatment of the ‘rapid’ line with the ATP depletion drugs 2‐deoxyglucose (2‐DG) and antimycin A resulted in the rapid inhibition of apical tip growth and a major [ATP]_i_ reduction within 10 min. This treatment induces much slower cytosolic acidification compared with SI responses, whereas F‐actin remodelling occurs with actin aggregation much later and only after [pH]_cyt_ reached 5.8. This suggests that the decrease in cytosolic pH may play a more predominant role in actin reorganization than ATP depletion. Overall, based on the timing of SI‐induced ATP depletion, cytosolic acidification and actin remodelling in the pollen tube of *P. rhoeas* and *A. thaliana* ‘rapid’ and ‘slow’ lines, the ATP depletion occurs slightly earlier than cytosolic acidification, indicating that ATP depletion may contribute to the decrease in [pH]_cyt_ after SI. This is further supported by the effect of ATP depletion drugs on [pH]_cyt_, although other factors are likely to be involved in orchestrating the significant cytosolic acidification and F‐actin reorganization during the SI response.

Animal cells subjected to severe ATP depletion die by necrosis; cells with less ATP depletion undergo apoptosis, which requires energy (Lieberthal *et al*., 1998). ATP depletion has been observed during various forms of PCD in plants and it has been suggested that intracellular energy depletion is an early signal that triggers PCD (Van Aken & Pogson, 2017). Self‐incompatibility results in a rapid drop of both [ATP]_i_ and [pH]_cyt_ and the formation of actin foci before PCD. We previously showed that artificial actin stabilization can trigger PCD (Thomas *et al*., 2006). In ATP‐depleted animal cells, actin aggregation is thought to be an attempt at ‘rescue’ during cellular stress to reduce ATP consumption (Bernstein & Bamburg, 2003; Atkinson *et al*., 2004; Xu & Bretscher, 2014). The F‐actin aggregation observed in ATP‐depleted pollen tubes (Fig. 9) may reflect a similar scenario. Biophysical *in vitro* studies show that spontaneous actin nucleation is maximal at pH 5.8 *in vitro* (Zimmerle & Frieden, 1988; Crevenna *et al*., 2013). As this is the threshold pH_cyt_ ‘tipping point’ for actin foci formation, this suggests that the extreme pH_cyt_ reduction triggered by ATP depletion contributes to the formation of these actin foci. This is supported by the finding that the ATP depletion drugs resulted in the rapid inhibition of apical tip growth and major [ATP]_i_ reduction within 10 min, but actin foci appeared much later and only when [pH]_cyt_ reached 5.8 (Fig. 9). Together, our data suggest that ATP depletion itself does not play a direct role in actin aggregation, and other factors (e.g. increases in [Ca^2+^]_cyt_ and ROS, and decreases in [pH]_cyt_) which we showed previously orchestrate alterations in actin configuration during SI, in concert with actin binding proteins (Snowman *et al*., 2002; Wilkins *et al*., 2015; Haque *et al*., 2020), are involved. A cartoon (Fig. 9) helps place the key *Papaver* SI‐induced events and their relative timings in context. Although SI culminates in PCD of incompatible pollen tubes, this occurs much later. This places ATP depletion and acidosis upstream of SI‐PCD (Fig. 9). As incompatible pollen tubes exhibit increased caspase‐3‐like/DEVDase activity needed for executing PCD several hours after SI induction, our observations show that they have sufficient ATP for enzyme activities and are still alive long after ATP depletion. This suggests that SI‐PCD requires energy and that pollen tubes obtain sufficient energy to remain alive for some time, allowing execution of later PCD processes.

In summary, here we have identified that SI triggers ATP depletion. Examining the possibility of other regulators involved in SI‐mediated alterations of actin organization, our studies have uncovered a link between ATP depletion and the extreme acidosis observed in incompatible pollen tubes. Both can affect the actin cytoskeleton organization. Our data suggest that cellular ATP levels and [pH]_cyt_ both play a pivotal role in mediating SI.

## Author contributions

LW, MB and VEF‐T designed the study; LW performed the research and analyzed data; AG‐K set up the *in vitro* actin slide assays; ZL and JC contributed constructs; and VEF‐T, LW, MB and JMD wrote the manuscript with input from all the other authors.

## Supporting information


**Fig. S1** Verification of [pH]_cyt_ values from calibration of the pH indicator pHGFP *in vivo* using propionic acid by comparison with those from a nigericin clamp‐based calibration.
**Fig. S2** Live‐cell imaging shows key features of filamentous (F)‐actin alterations triggered by self‐incompatibility in *Arabidopsis thaliana* transgenic lines co‐expressing PrpS_1_ and Lifeact‐mRuby2.
**Fig. S3** Representative time‐lapse images and corresponding skeletonized actin structures in a pollen tube from a transgenic *Arabidopsis thaliana* line co‐expressing PrpS_1_ and Lifeact‐mRuby2 undergoing a ‘rapid’ self‐incompatibility response.
**Fig. S4** Representative images of altered filamentous (F)‐actin organization in the pollen tube categorized into four stages after self‐incompatibility induction in an *Arabidopsis thaliana* ‘slow’ line co‐expressing both PrpS_1_‐GFP and Lifeact‐mRuby2.
**Fig. S5** Visualization of the dynamics of self‐incompatibility‐induced filamentous (F)‐actin foci formation in pollen tubes from a ‘rapid’ *Arabidopsis thaliana* line.
**Fig. S6** Timing of pollen tube growth arrest after ATP depletion.
**Fig. S7** Pollen tubes do not exhibit elevated caspase‐3‐like/DEVDase activity during the self‐incompatibility‐ or ATP‐depletion induced acidification time‐period.
**Table S1** Details of the transgenic *Arabidopsis thaliana* self‐incompatible‐lines used in this study.
**Table S2** Propidium iodide (PI)‐staining of *Arabidopsis thaliana* pollen tubes from the ‘rapid’ line co‐expressing PrpS_1_ and Lifeact‐mRuby2 after self‐incompatibility induction.
**Table S3** Quantification of cytosolic pH ([pH]_cyt_) and corresponding proton concentration in different distal areas of growing and self‐incompatibility‐induced *Arabidopsis thaliana* pollen tubes co‐expressing PrpS_1_ and pHGFP.

**Video S1** Time‐lapse image series of a pollen tube from a ‘rapid’ *Arabidopsis thaliana* line co‐expressing PrpS_1_ and Lifeact‐mRuby2 after self‐incompatibility‐induction showing filamentous (F)‐actin reorganization and foci formation.

**Video S2** Time‐lapse image series of a representative pollen tube from a ‘rapid’ *Arabidopsis thaliana* line after treatment with 2‐deoxyglucose (2‐DG) and antimycin A, showing timing of growth arrest, which is a key feature of self‐incompatibility.

**Video S3** Time‐lapse ratio‐image series of a normally growing pollen tube from a ‘rapid’ *Arabidopsis thaliana* line co‐expressing PrpS_1_, pHGFP and Lifeact‐mRuby2 showing the distribution of cytosolic pH ([pH]_cyt_) during normal growth.

**Video S4** Time‐lapse ratio‐image series of a pollen tube from a ‘rapid’ *Arabidopsis thaliana* line co‐expressing PrpS_1_, pHGFP and Lifeact‐mRuby2 after self‐incompatibility‐induction, showing spatiotemporal changes in cytosolic pH ([pH]_cyt_) during the SI response.
Please note: Wiley Blackwell are not responsible for the content or functionality of any Supporting Information supplied by the authors. Any queries (other than missing material) should be directed to the *New Phytologist* Central Office.

## Data Availability

The data that support the findings of this study are available from the corresponding author upon reasonable request.
