# Supplementary material for: ATP depletion plays a pivotal role in self‐incompatibility, revealing a link between cellular energy status, cytosolic acidification and actin remodelling in pollen tubes
Source: New Phytol. 2022 Jul 23;236(5):1691–707. doi: 10.1111/nph.18350 (PMC9796540; doi:10.1111/nph.18350)
Supplement: Supplementary file 1 — Fig. S1 Verification of [pH]cyt values from calibration of the pH indicator pHGFP in vivo using propionic acid by comparison with those from a nigericin clamp‐based calibration. Fig. S2 Live‐cell imaging shows key features of filamentous (F)‐actin alterations triggered by self‐incompatibility in Arabidopsis thaliana transgenic lines co‐expressing PrpS1 and Lifeact‐mRuby2. Fig. S3 Representative time‐lapse images and corresponding skeletonized actin structures in a pollen tube from a transgenic Arabidopsis thaliana line co‐expressing PrpS1 and Lifeact‐mRuby2 undergoing a ‘rapid’ self‐incompatibility response. Fig. S4 Representative images of altered filamentous (F)‐actin organization in the pollen tube categorized into four stages after self‐incompatibility induction in an Arabidopsis thaliana ‘slow’ line co‐expressing both PrpS1‐GFP and Lifeact‐mRuby2. Fig. S5 Visualization of the dynamics of self‐incompatibility‐induced filamentous (F)‐actin foci formation in pollen tubes from a ‘rapid’ Arabidopsis thaliana line. Fig. S6 Timing of pollen tube growth arrest after ATP depletion. Fig. S7 Pollen tubes do not exhibit elevated caspase‐3‐like/DEVDase activity during the self‐incompatibility‐ or ATP‐depletion induced acidification time‐period. Table S1 Details of the transgenic Arabidopsis thaliana self‐incompatible‐lines used in this study. Table S2 Propidium iodide (PI)‐staining of Arabidopsis thaliana pollen tubes from the ‘rapid’ line co‐expressing PrpS1 and Lifeact‐mRuby2 after self‐incompatibility induction. Table S3 Quantification of cytosolic pH ([pH]cyt) and corresponding proton concentration in different distal areas of growing and self‐incompatibility‐induced Arabidopsis thaliana pollen tubes co‐expressing PrpS1 and pHGFP. [file NPH-236-1691-s004.pdf]

New Phytologist

New Phytologist **Supporting Information**

Article title: **ATP depletion plays a pivotal role in self-incompatibility, revealing a link between cellular energy status, cytosolic acidification and actin remodelling in pollen tubes**

Authors: **Ludi Wang, Zongcheng Lin, José Carli, Agnieszka Gladala-Kostarz, Julia M. Davies, Vernonica E. Franklin-Tong, Maurice Bosch**

Article acceptance date: 15 June 2022

The following Supporting Information is available for this article:

Fig. S1 Verification of $[pH]_{cyt}$ values from calibration of the pH indicator pHGFP *in vivo* using propionic acid by comparison with those from a nigericin clamp-based calibration.

Fig. S2 Live-cell imaging shows key features of filamentous (F)-actin alterations triggered by self-incompatibility (SI) in *Arabidopsis thaliana* transgenic lines co-expressing $PrpS_1$ and Lifeact-mRuby2.

Fig. S3 Representative time-lapse images and corresponding skeletonized actin structures in a pollen tube from a transgenic *Arabidopsis thaliana* line co-expressing $PrpS_1$ and Lifeact-mRuby2 undergoing a 'rapid' self-incompatibility (SI) response.

Fig. S4 Representative images of altered filamentous (F)-actin organization in the pollen tube categorized into four stages after self-incompatibility (SI) induction in an *Arabidopsis thaliana* 'slow' line co-expressing both $PrpS_1$-GFP and Lifeact-mRuby2.

Fig. S5 Visualization of the dynamics of self-incompatibility (SI)-induced filamentous (F)-actin foci formation in pollen tubes from a 'rapid' *Arabidopsis thaliana* line.

Fig. S6 Timing of pollen tube growth arrest after ATP depletion.

Fig. S7 Pollen tubes do not exhibit elevated caspase-3-like/DEVDase activity during the self-incompatibility (SI)- or ATP-depletion induced acidification time-period.

Table S1 Details of the transgenic *Arabidopsis thaliana* self-incompatible (SI)-lines used in this

study.

Table S2 Propidium iodide (PI)-staining of *Arabidopsis thaliana* pollen tubes from the 'rapid' line co-expressing $PrpS_1$ and Lifeact-mRuby2 after self-incompatibility (SI) induction.

Table S3 Quantification of cytosolic pH ($[pH]_{cyt}$) and corresponding proton concentration in different distal areas of growing and self-incompatibility (SI)-induced *Arabidopsis thaliana* pollen tubes co-expressing $PrpS_1$ and pHGFP.

Video S1 Time-lapse image series of a pollen tube from a 'rapid' *Arabidopsis thaliana* line co-expressing $PrpS_1$ and Lifeact-mRuby2 after self-incompatibility (SI)-induction showing filamentous (F)-actin reorganization and foci formation.

Video S2 Time-lapse image series of a representative pollen tube from a 'rapid' *Arabidopsis thaliana* line after treatment with 2-deoxyglucose (2-DG) and antimycin A, showing timing of growth arrest, which is a key feature of self-incompatibility (SI).

Video S3 Time-lapse ratio-image series of a normally growing pollen tube from a 'rapid' *Arabidopsis thaliana* line co-expressing $PrpS_1$, pHGFP and Lifeact-mRuby2 showing the distribution of cytosolic pH ($[pH]_{cyt}$) during normal growth.

Video S4 Time-lapse ratio-image series of a pollen tube from a 'rapid' *Arabidopsis thaliana* line co-expressing $PrpS_1$, pHGFP and Lifeact-mRuby2 after self-incompatibility (SI)-induction, showing spatiotemporal changes in cytosolic pH ($[pH]_{cyt}$) during the SI response.

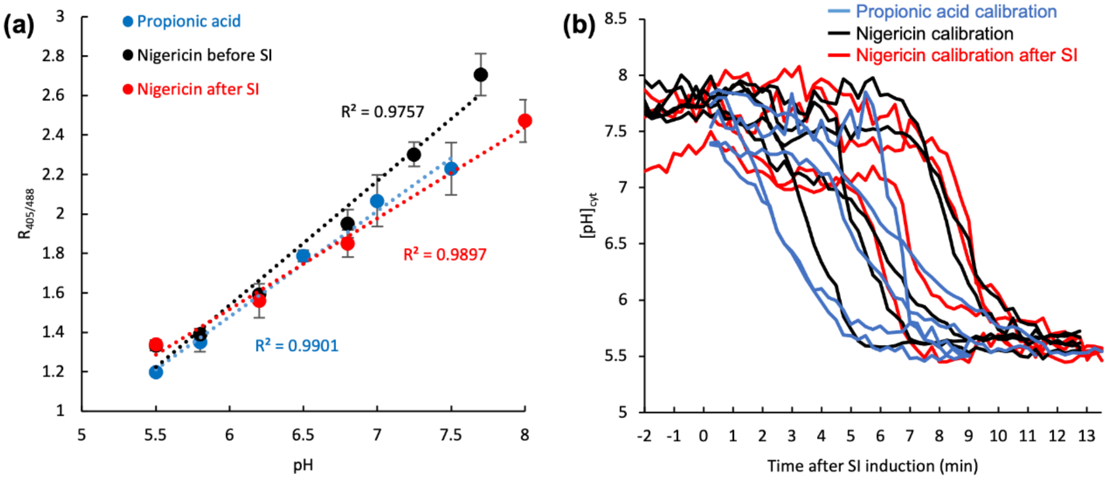

Fig. S1 Verification of $[pH]_{cyt}$ values from calibration of the pH indicator pHGFP *in vivo* using propionic acid by comparison with those from a nigericin clamp-based calibration. All the *in vivo* $[pH]_{cyt}$ measurements presented in this paper were made using propionic acid calibration. To validate the calibration of the *in vivo* $[pH]_{cyt}$ measurements made using propionic acid, we subsequently generated calibration curves using nigericin clamping according to Hoffmann *et al.* (2020) both on untreated pollen tubes and after self-incompatibility (SI) treatment. Comparison between the different calibration methods show the validity of the propionic acid calibration. **(a)** Comparison of representative calibration curves of pHGFP made using *Arabidopsis thaliana* pollen tubes growing *in vitro* with 50 mM propionic acid (blue, n = 5), nigericin clamping (30 µM nigericin and 140 mM KCl in 10 mM sodium phosphate buffer of different pH) before SI induction (black, n = 5) and 15-20 min after SI induction (red, n = 4). Error bars indicate ± SD. Propionic acid and nigericin clamping calibrations yielded similar ratio values over the 5.5 to 7 pH range. **(b)** Representative examples of $[pH]_{cyt}$ measured in *Arabidopsis thaliana* pollen tubes after the SI response with $[pH]_{cyt}$ calibration using the three methods described in (a). The curves show very similar extreme acidification responses within a few minutes of SI induction, plateauing around pH 5.5, regardless of the calibration method. All pollen tubes used for (a) and (b) were Arabidopsis lines expressing PrpS (*pntp303::pHGFP_pntp303::PrpS$_1$, pntp303::Lifeact-mRuby2*). Together these calibration curves verify the $[pH]_{cyt}$ values reported in the manuscript using the *in vivo* propionic acid method. They also support the previous reports of SI-induced cytosolic acidification to pH 5.5. Bosch *et al.* (2007) imaged $[pH]_{cyt}$ using BCECF and reported a $[pH]_{cyt}$ of 5.5 after SI, using an *in vitro* calibration curve according to Feijo *et*

al. (1999). Wilkins *et al.* (2015) used a similar *in vitro* protocol, with slight changes, and also obtained a $[pH]_{cyt}$ of 5.5 after SI. In this paper we performed *in vivo* calibrations in pollen tubes, using propionic acid buffer to manipulate the $[pH]_{cyt}$ in pollen tubes expressing pHGFP. SI-induced acidification of $[pH]_{cyt}$ to 5.5 is conserved regardless of reporter or calibration method.

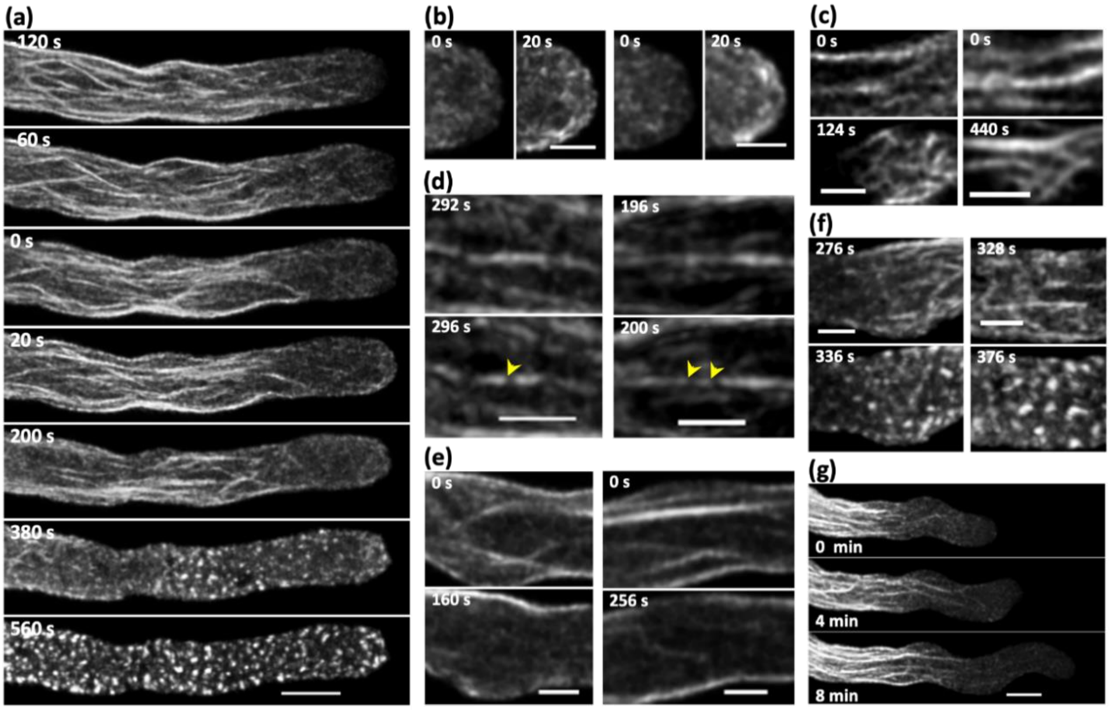

Fig. S2 Live-cell imaging shows key features of filamentous (F)-actin alterations triggered by self-incompatibility (SI) in *Arabidopsis thaliana* transgenic lines co-expressing $PrpS_1$ and Lifeact-mRuby2. The Arabidopsis 'rapid' line expressing both $PrpS_1$ and Lifeact-mRuby2 and confocal imaging was used to follow alterations in actin triggered by SI. Time stamps indicate time after SI induction. **(a)** Max-projection time-lapse images of actin cytoskeleton in a representative pollen tube before and after SI induction. '0 s' indicates the moment of SI induction. Scale bar, 5 µm. **(b)** Two representative pairs of time-lapse max-projection images showing little detectable F-actin in the apical region prior to SI (0 s) and actin accumulation at the tip 20 sec after SI induction. Scale bar, 2 µm. **(c)** Two representative image pairs showing reorientation of F-actin bundles in the cortical area (28-35 µm from tip) during the early SI response. Scale bar, 2 µm. **(d)** Two representative examples of actin filament bundles undergoing fragmentation during the early stage of SI response (mid-plane optical sections of two image pairs). Arrow heads indicate fragmentation of actin bundles. Scale bar, 2 µm. **(e)** Two Image pairs showing apparent F-actin depolymerization in the shank region after SI; mid-plane optical sections. Scale bar, 2 µm. **(f)** Two image pairs highlighting the actin foci formation after SI induction (max projections); scale bar, 2 µm. **(g)** Representative images of a pollen tube undergoing a compatible interaction with recombinant $PrsS_3$ showing no actin alterations and the pollen tube continues to grow. This control demonstrates that the actin alterations observed in these lines are *S*-specific. Scale bar, 5 µm.

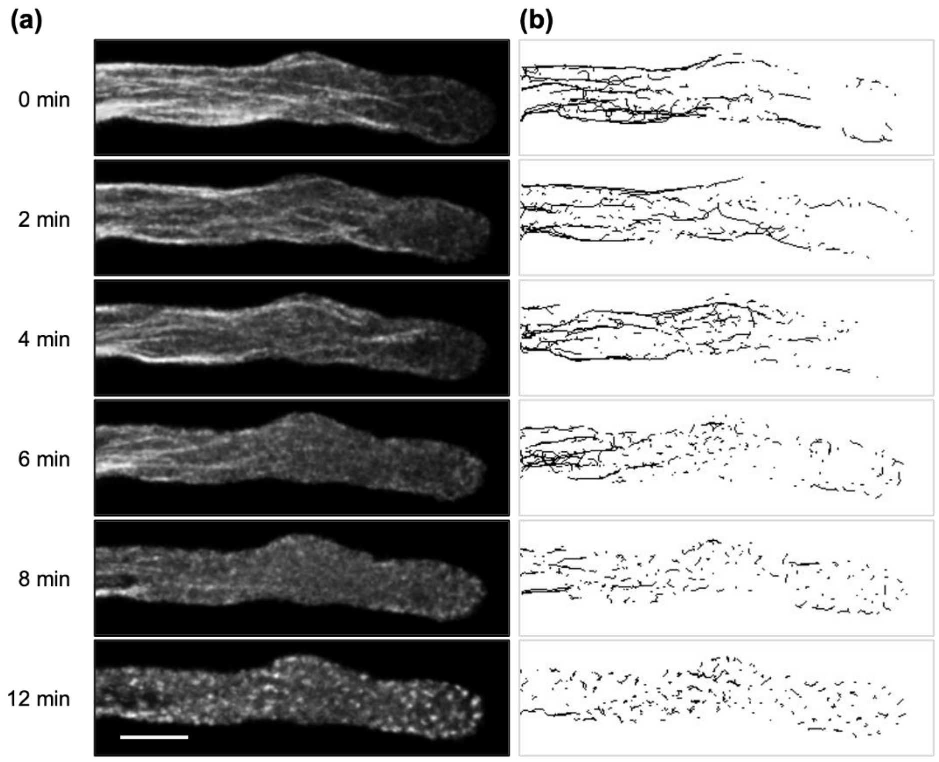

Fig. S3 Representative time-lapse images and corresponding skeletonized actin structures in a pollen tube from a transgenic *Arabidopsis thaliana* line co-expressing PrpS$_1$ and Lifeact-mRuby2 undergoing a 'rapid' self-incompatibility (SI) response. (a) Fluorescence microscopy images showing F-actin decorated by Lifeact-mRuby2. Scale bar, 5 µm. **(b)** Skeletonised images of F-actin structures in (a).

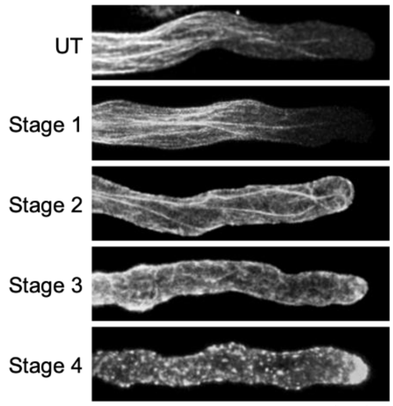

Fig. S4 Representative images of altered filamentous (F)-actin organization in the pollen tube categorized into four stages after self-incompatibility (SI) induction in an *Arabidopsis thaliana* 'slow' line co-expressing both PrpS$_1$-GFP and Lifeact-mRuby2. Although the timing of the actin alteration was different in the 'rapid' and 'slow' lines, the overall sequence and characteristics were very similar. We therefore identified four key 'stages' as categories that were typical of the alterations observed. We numbered the different stages to allow comparison between events observed in the 'slow' lines, as there was considerable variation in timing of the response. Stage 1 looked essentially like untreated pollen tubes. At stage 2, long longitudinal F-actin bundles/cables were still apparent in the central region, but the cortical region had short F-actin filaments that were no longer aligned with the growth axis. At stage 3, very few intact actin filament cables were observed. Stage 4 had large punctate actin foci. UT, untreated. Scale bar, 5 µm.

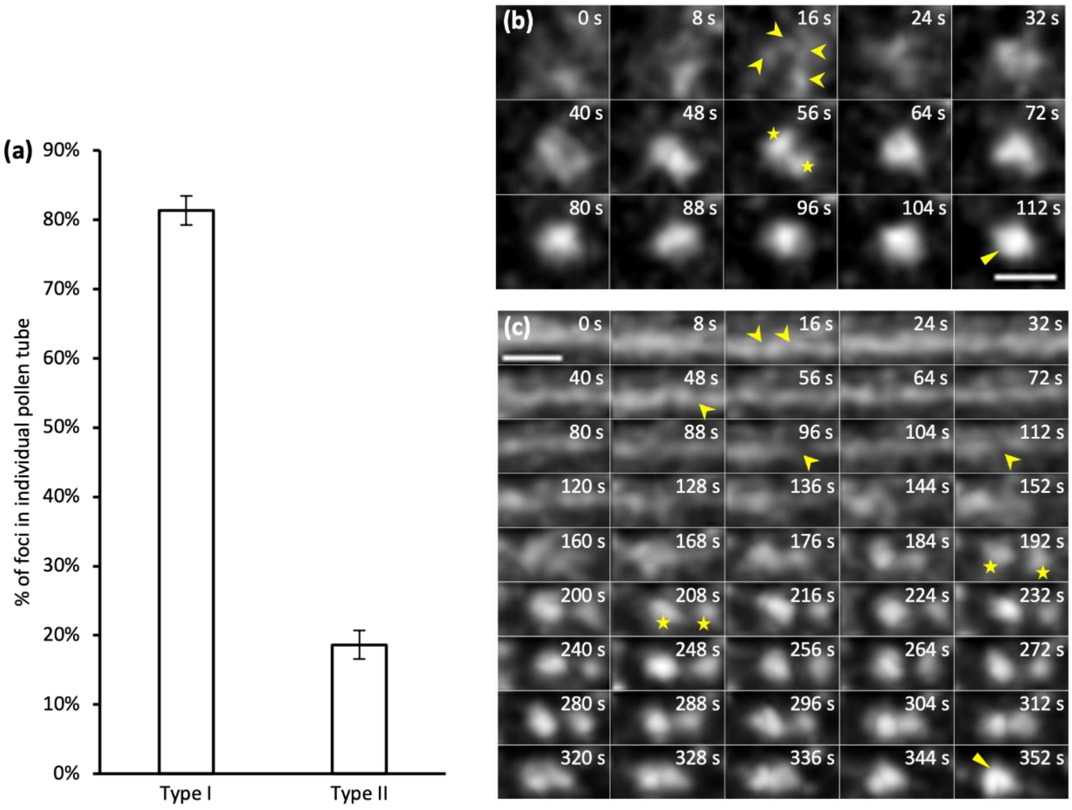

Fig. S5 Visualization of the dynamics of self-incompatibility (SI)-induced filamentous (F)-actin foci formation in pollen tubes from a 'rapid' *Arabidopsis thaliana* line. (a) Quantification of F-actin foci that were formed following two different types of progression. Type I: Foci formed from short randomly orientated F-actin fragments that aggregated relatively rapidly; Type II: Foci formed directly from severed actin filament cables, with fragments of actin bundles aggregating to form the distinctive large foci, or with small foci subsequently coalescing to form larger foci. Error bars indicate ± SD. More than 80 foci were categorised per pollen tube (over 12 pollen tubes undergoing the SI response were analysed in total). **(b)** and **(c)** Two further representative examples of time-lapse images of the formation of SI-induced punctate actin foci. '0 s' indicates the start time-point of selected time-lapse images. Scale bar, 1 µm. **(b)** Time-lapse images showing formation of F-actin foci during 6 min 4 s – 7 min 56 s (time stamps 0-112 s) after SI induction with a Type I progression. Short actin fragments (arrow heads) appeared to aggregate (indicated by a pair of stars) and later to form a larger punctate structure (triangle). **(c)** Time-lapse images demonstrating formation of F-actin foci during 2 min 48 s – 8 min 40 s (time stamps 0-352 s) after SI induction with a Type II progression. Fragmentation of a thick actin filament bundle (arrow heads) was followed by aggregation of fragments (indicated by pairs of stars) into a larger punctate

structure (triangle).

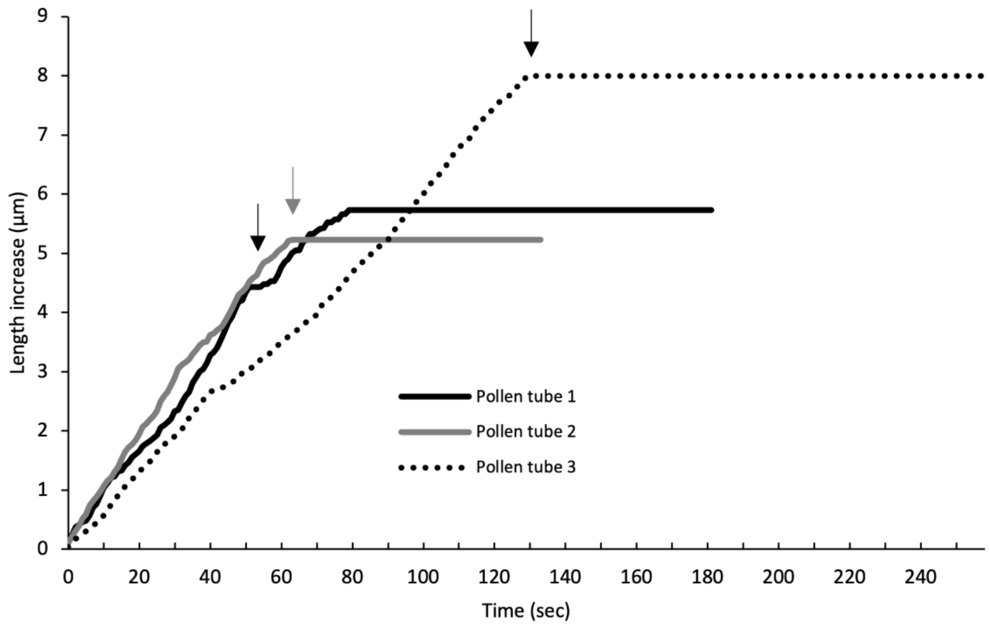

Fig. S6 Timing of pollen tube growth arrest after ATP depletion. Plots showing pollen tube growth and the timing of growth arrest, which is a key feature of self-incompatibility (SI). The growth of three representative pollen tubes is shown before and after ATP depletion using 10 µM antimycin A and 15 mM 2-deoxyglucose (2-DG). Arrows indicate the timepoints when treatment was added. As expected, the ATP depletion drugs led to almost instantaneous growth arrest, within 1 min.

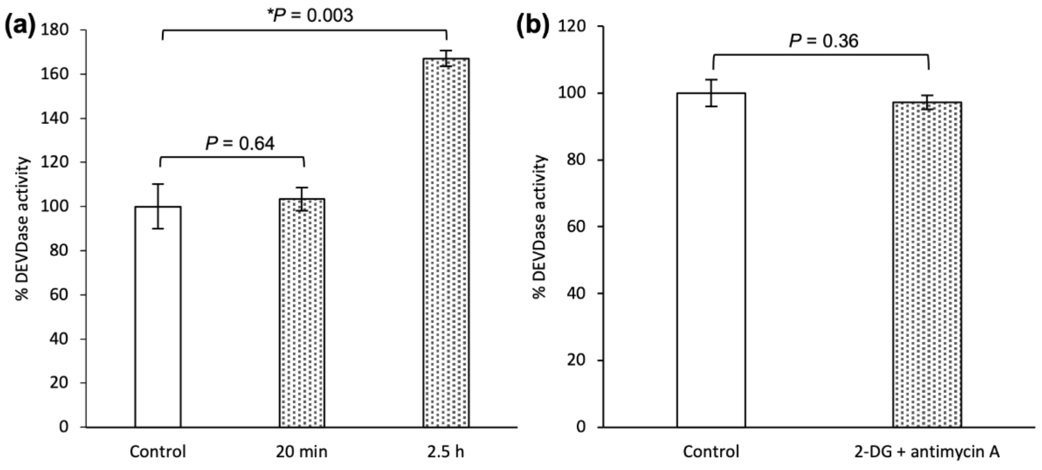

Fig. S7 Pollen tubes do not exhibit elevated caspase-3-like/DEVDase activity during the self-incompatibility (SI)- or ATP-depletion induced acidification time-period. (a) Caspase-3/DEVDase activity in pollen tube extracts from an Arabidopsis 'rapid' line (*pntp303::pHGFP_pntp303::PrpS*$_1$) after SI induction. 100% activity represents zero (baseline) activity in unchallenged pollen tubes. No significant increase in DEVDase activity was observed 20 min after SI induction in Arabidopsis pollen tubes compared to normally growing control pollen tubes (with growth medium (GM) added), while after 2.5 hours of SI induction, a significant increase in DEVDase activity was detected (n = 3). Error bars indicate ± SD. This provides evidence that SI-induced pollen tubes are not dying during the acidification period. **(b)** Caspase-3/DEVDase activity in Arabidopsis pollen tube extracts treated with ATP depletion drugs. 100% activity represents zero (baseline) activity in unchallenged pollen tubes. Five hours after addition of the ATP depletion drugs (2-deoxyglucose (2-DG) + antimycin A) to pollen tubes of an Arabidopsis 'rapid' line, way after the time frame of when the ATP depletion was observed, there was no evidence of caspase-3/DEVDase activity detected (n = 3). After 5 h treatment with these two drugs together, Arabidopsis pollen tubes had caspase-3/DEVDase activity that was not significantly different from control, normally growing pollen tubes that were treated with GM. Error bars indicate ± SD. This provides evidence that artificial ATP depletion using these drugs does not trigger caspase-3/DEVDase activity, even long after the time-frame when the ATP depletion was observed.

Table S1 Details of the transgenic *Arabidopsis thaliana* self-incompatible (SI)-lines used in this study. Two types of transgenic Arabidopsis lines with different levels of expression of PrpS in the pollen (see Wang *et al*. 2020) were used in this study. The 'rapid' lines express a relatively high level of PrpS and exhibit a fast SI response. For example, most pollen tubes from a 'rapid' line exhibited punctate foci within 10 minutes of SI induction, while this would normally take one hour in *Papaver* pollen tubes using a similar concentration of PrsS recombinant protein. The 'rapid' line was therefore useful for visualizing SI-induced alterations continuously from start to finish in a single pollen tube, which would otherwise be impractical with normal expression levels of PrpS. The 'slow' line expresses relatively low level of PrpS and has a slower SI response. Most pollen tubes from this line exhibited punctate foci after 1-1.5 hours of SI induction. This was useful to study the shortening rate of actin filament cables induced by SI. The genetically encoded actin probe Lifeact-mRuby2 (Riedl *et al*., 2008; Bascom *et al*., 2018) and pHGFP (Moseyko and Feldman, 2001) were co-expressed in the 'rapid' line 3 to enable imaging of F-actin and $[pH]_{cyt}$ after SI.

Lines	Constructs	Corresponding figures
1 (Rapid)	*pntp303::YC3.6-**pntp303::PrpS$_1$**, pntp303::Lifeact-mRuby2* (Wang *et al*., 2020)	Figure 1a-c, 1f, S2, S3, S5 Video S1
2 (Slow)	*pntp303::Lifeact-mRuby2, **pntp303::PrpS$_1$-GFP*** (de Graaf *et al*., 2012; Wang *et al*., 2020)	Figure 1d-e, 2b, 5d-e, 6e, S4
3 (Rapid)	*pntp303::pHGFP-**pntp303::PrpS$_1$**, pntp303::Lifeact-mRuby2* (Wang *et al*., 2020)	Figure 2a, 2d-e, 3, 4, 6a-d, 7, 8a-b, S1, S6, S7, Video S2, S3, S4, Table S2, S3
4	***pUBQ10::PrpS$_1$-GFP*** (Lin *et al*., 2020)	Figure 8c-f

Table S2 Propidium iodide (PI)-staining of *Arabidopsis thaliana* pollen tubes from the 'rapid' line co-expressing $PrpS_1$ and Lifeact-mRuby2 after self-incompatibility (SI) induction. None of the pollen tubes observed (n = 1338 total) had PI-stained nuclei at any time point up to 30 min after SI induction, which was when cellular $[ATP]_i$ and $[pH]_{cyt}$ had already decreased and plateaued. At 60 min after SI induction only ~10 % of pollen tubes were PI-positive, but by 3 h after SI-induction 96% (n = 265) of observed pollen tubes were PI-positive (n = 265 pollen tubes observed for 180 min). This demonstrates that no permeabilization of the plasma membrane was observed within the time-frame of the significant $[pH]_{cyt}$ decrease observed in these studies. This provides evidence that the SI-induced pollen tubes were not dying at this early stage of SI and membrane integrity was lost much later. These data provide good evidence that cytosolic acidification occurred upstream of SI-induced programmed cell death (PCD) rather than being merely a consequence of PCD.

	Numbers of pollen tubes with PI-stained/ non-stained nuclei				
Time after SI (min)	Sample 1	Sample 2	Sample 3	Sample 4	**% Mean ± SD**
0	0/60	0/72	0/50	0/50	**0.0 ± 0.0**
5	0/57	0/62	0/73	0/50	**0.0 ± 0.0**
10	0/74	0/61	0/67	0/86	**0.0 ± 0.0**
20	0/73	0/86	0/66	0/63	**0.0 ± 0.0**
30	0/76	0/89	0/59	0/64	**0.0 ± 0.0**
60	7/51	4/56	5/47	7/52	**9.6 ± 2.2**
180	78/3	69/4	50/2	59/5	**96.3 ± 1.6**

Table S3 Quantification of cytosolic pH ([pH]$_{cyt}$) and corresponding proton concentration in different distal areas of growing and self-incompatibility (SI)-induced *Arabidopsis thaliana* pollen tubes co-expressing PrpS$_1$ and pHGFP. (a) Quantification of the [pH]$_{cyt}$ in four distal areas of growing Arabidopsis pollen tubes. n = 3. **(b)** Quantification of the [pH]$_{cyt}$ in four distal areas of pollen tubes after SI induction. n = 3. **(c)** Proton concentrations (µM) corresponding to the pH values in (a). **(d)** Proton concentrations (µM) corresponding to the pH values in (b). ± values indicate SD.

(a)

	Distance from apex (µm)			
**Time**	**0-3**	**3-15**	**15-25**	**25-35**
**0 s**	6.76 ± 0.08	6.93 ± 0.22	7.22 ± 0.21	7.40 ± 0.14
**30 s**	6.80 ± 0.09	7.10 ± 0.21	7.25 ± 0.03	7.40 ± 0.07
**60 s**	6.90 ± 0.16	7.06 ± 0.22	7.34 ± 0.16	7.44 ± 0.11
**90 s**	6.81 ± 0.16	7.03 ± 0.16	7.15 ± 0.16	7.31 ± 0.11
**120 s**	6.65 ± 0.17	7.06 ± 0.14	7.21 ± 0.23	7.33 ± 0.22

(b)

	Distance from apex (µm)			
**Time**	**0-3**	**3-15**	**15-25**	**25-35**
**0 min**	6.86 ± 0.33	7.00 ± 0.24	7.15 ± 0.29	7.50 ± 0.18
**2 min**	6.38 ± 0.63	6.40 ± 0.59	6.54 ± 0.60	6.70 ± 0.61
**3 min**	6.00 ± 0.42	6.07 ± 0.40	6.13 ± 0.43	6.23 ± 0.41
**4 min**	5.65 ± 0.39	5.65 ± 0.30	5.66 ± 0.29	5.75 ± 0.30

(c)

	Distance from apex (µm)			
**Time**	**0-3**	**3-15**	**15-25**	**25-35**
**0 s**	0.17 ± 0.03	0.13 ± 0.05	0.06 ± 0.03	0.04 ± 0.01
**30 s**	0.16 ± 0.04	0.09 ± 0.04	0.06 ± 0.00	0.04 ± 0.01
**60 s**	0.13 ± 0.05	0.10 ± 0.05	0.05 ± 0.02	0.04 ± 0.01
**90 s**	0.16 ± 0.05	0.10 ± 0.04	0.07 ± 0.03	0.05 ± 0.01
**120 s**	0.24 ± 0.10	0.09 ± 0.03	0.07 ± 0.04	0.05 ± 0.03

(d)

	Distance from apex (µm)			
**Time**	**0-3**	**3-15**	**15-25**	**25-35**
**0 min**	0.17 ± 0.13	0.11 ± 0.07	0.08 ± 0.06	0.03 ± 0.01
**2 min**	0.67 ± 0.51	0.61 ± 0.46	0.45 ± 0.35	0.33 ± 0.34
**3 min**	1.28 ± 0.86	1.08 ± 0.71	0.97 ± 0.66	0.81 ± 0.76
**4 min**	2.78 ± 1.71	2.56 ± 1.35	2.46 ± 1.27	2.04 ±1.25

Video S1 Time-lapse image series of a pollen tube from a 'rapid' *Arabidopsis thaliana* line co-expressing $PrpS_1$ and Lifeact-mRuby2 after self-incompatibility (SI)-induction showing filamentous (F)-actin reorganization and foci formation. Confocal imaging of a representative pollen tube expressing both $PrpS_1$ and Lifeact-mRuby2 was used to follow alterations in actin triggered by SI. Time stamps indicate time after SI induction. Note the striking aggregation of actin fragments at ~ 6 min.

Video S2 Time-lapse image series of a representative pollen tube from a 'rapid' *Arabidopsis thaliana* line after treatment with 2-deoxyglucose (2-DG) and antimycin A, showing timing of growth arrest, which is a key feature of self-incompatibility (SI). As expected, the ATP depletion drugs led to almost instantaneous growth arrest, within 1 min, a similar time-frame as observed for SI in the rapid Arabidopsis SI-line.

Video S3 Time-lapse ratio-image series of a normally growing pollen tube from a 'rapid' *Arabidopsis thaliana* line co-expressing $PrpS_1$, pHGFP and Lifeact-mRuby2 showing the distribution of cytosolic pH ($[pH]_{cyt}$) during normal growth. Confocal imaging of pollen tubes expressing both $PrpS_1$ and pHGFP was used to follow alterations in $[pH]_{cyt}$ in a representative normally growing pollen tube. The pseudocolour shows pH values, with 'hot' colours indicating high pH and 'cool' colours indicating low pH. Overall, the cytosolic pH is ~neutral, with conspicuous peripheral alkaline patches.

Video S4 Time-lapse ratio-image series of a pollen tube from a 'rapid' *Arabidopsis thaliana* line co-expressing $PrpS_1$, pHGFP and Lifeact-mRuby2 after self-incompatibility (SI)-induction, showing spatiotemporal changes in cytosolic pH ($[pH]_{cyt}$) during the SI response. Confocal imaging of a representative pollen tube expressing both $PrpS_1$ and pHGFP was used to follow alterations in $[pH]_{cyt}$ triggered by SI. Time stamps indicate time after SI induction. The pseudocolour shows pH values, with 'hot' colours indicating high pH and 'cool' colours indicating low pH. Note the peripheral acidic patches appearing at ~2-3 min and subsequent cytosolic acidification.

REFERENCES

Bascom CS Jr, Winship LJ, Bezanilla M. 2018. Simultaneous imaging and functional studies reveal a tight correlation between calcium and actin networks. *Proceedings of the National Academy of Sciences, USA* **115**: E2869–E2878.

de Graaf BH, Vatovec S, Juárez-Díaz JA, Chai L, Kooblall K, Wilkins KA, Zou H, Forbes T, Franklin FC, Franklin-Tong VE. 2012. The *Papaver* self-Incompatibility pollen *S*-Determinant, PrpS, functions in *Arabidopsis thaliana*. *Current Biology* **22**: 154-159.

Feijó JA, Sainhas J, Hackett GR, Kunkel JG, Hepler PK. 1999. Growing pollen tubes possess a constitutive alkaline band in the clear zone and a growth-dependent acidic tip. *Journal of Cell Biology* **144**: 483-496.

Hoffmann RD, Portes MT, Olsen LI, Damineli DS, Hayashi M, Nunes CO, Pedersen JT, Lima PT, Campos C, Feijó JA, Palmgren M. 2020. Plasma membrane H^+-ATPases sustain pollen tube growth and fertilization. *Nature Communications* **11**: 2395.

Lin Z, Xie F, Triviño M, Karimi M, Bosch M, Franklin-Tong VE, Nowack MK. 2020. Ectopic expression of a self-incompatibility module triggers growth arrest and cell death in vegetative cells. *Plant Physiology* **183**: 1765-1779.

Moseyko N, Feldman LJ. 2001. Expression of pH-sensitive green fluorescent protein in *Arabidopsis thaliana*. *Plant, Cell & Environment* **24**: 557–563.

Riedl J, Crevenna AH, Kessenbrock K, Yu JH, Neukirchen D, Bista M, Bradke F, Jenne D, Holak TA, Werb Z, et al. 2008. Lifeact: a versatile marker to visualize F-actin. *Nature Methods* **5**: 605–607.

Wang L, Triviño M, Lin Z, Carli J, Eaves DJ, Van Damme D, Nowack MK, Franklin-Tong VE, Bosch M. 2020. New opportunities and insights into *Papaver* self-incompatibility by imaging engineered Arabidopsis pollen. *Journal of Experimental Botany* **71**: 2451-2463.

Wilkins KA, Bosch M, Haque T, Teng N, Poulter NS, Franklin-Tong VE. 2015. Self-incompatibility-induced programmed cell death in field poppy pollen involves dramatic acidification of the incompatible pollen tube cytosol. *Plant Physiology* **167**: 766-779.
